# Transcriptomic changes in the large organs in lethal meningococcal shock are reflected in a porcine shock model

**DOI:** 10.3389/fcimb.2022.908204

**Published:** 2022-08-11

**Authors:** Berit Sletbakk Brusletto, Bernt Christian Hellerud, Ole Kristoffer Olstad, Reidun Øvstebø, Petter Brandtzaeg

**Affiliations:** ^1^ Department of Medical Biochemistry, Oslo University Hospital, Oslo, Norway; ^2^ Institute of Immunology, Oslo University Hospital, Oslo, Norway; ^3^ Department of Pediatrics, Oslo University Hospital, Oslo, Norway; ^4^ Institute of Clinical Medicine, Faculty of Medicine, University of Oslo, Oslo, Norway

**Keywords:** *Neisseria meningitidis*, meningococcal septic shock, porcine shock model, organ specific transcriptional profile, IPA comparison analysis, disseminated intravascular coagulation (DIC)

## Abstract

**Background:**

Fulminant meningococcal sepsis with shock and multiple organ failure is associated with a massive systemic inflammatory response involving solid organs. We have previously established a porcine model of the disease to study pathophysiologic and possible therapeutic strategies.

**Objective:**

This study examined whether the organ specific gene expression profile in such a large animal model reflects the profile seen in patients with fulminant meningococcal sepsis.

**Patients and methods:**

Data from gene expression profiles induced in organs from patients (n=5) and the porcine model (n=8) were imported into the Ingenuity pathway analysis (IPA) software for comparison analysis. The number of meningococci in the organs were quantified by real time-PCR.

**Results:**

The all-over transcriptional activation between different organs revealed a striking concordance between the patients and the pigs regarding the pattern of transcriptional activation and activated pathways. Comparison analysis demonstrated similar pattern of upregulation of genes being associated with a large range of inflammatory biofunctions in the patients and the porcine model. Genes associated with biofunctions such as organismal death, morbidity and mortality were similarly downregulated in the patients and the porcine model. Comparison analysis of main predicted canonical pathways also demonstrated a high degree of similarity regarding up- and downregulation in both groups. Core analysis revealed different top-upstream regulators in the different organs in the patients. In the patients pro-inflammatory regulators were most activated in the lungs. In the other organs up-stream factors that regulate signaling pathways involved in development, growth, repair and homeostasis and triglyceride synthesis were most activated. In the porcine model, the top-upstream regulators were pro-inflammatory in all organs. The difference may reflect the shorter duration of the porcine experiment than the duration of the patient’s infection before death.

**Conclusion:**

The inflammatory responses measured on the transcriptomic level in organs in patients with fulminant meningococcal sepsis is reproduced in the porcine model of the disease, although some differences may exist regarding the top-upregulated factors in individual organs. Thus, this large animal model reproduces important immunological features of meningococcal sepsis and can be a valuable tool in further investigations of inflammatory aspects and possible treatment options

## Introduction

Shock and multiple organ failure are extreme disease manifestations induced by *Neisseria meningitidis*, a usually harmless bacterium residing in the upper airways (Brandtzaeg, 1995; de Kleijn et al., 1998; van Deuren et al., 2000; Rosenstein et al., 2001; Riordan et al., 2002; Brandtzaeg, 2006; Stephens et al., 2007; Brandtzaeg and van Deuren, 2012). The fulminant character of meningococcal septic shock and multiple organ failure is caused by the exceptional ability of certain clones of meningococci to proliferate in the blood and adjacent perivascular tissues, resulting in 10^6^ to 10^8^ bacteria per milliliter of plasma as determined by real-time PCR of the *ctr*A gene, which is present in one copy in the *N. meningitidis* genome (Hackett et al., 2002; Ovstebo et al., 2004; Darton et al., 2009; Brusletto et al., 2017). The unusually high levels of meningococcal DNA in the circulation and perivascular space are closely associated with levels of lipopolysaccharide (LPS), up to 3,000 endotoxin units per milliliter of plasma (Brandtzaeg et al., 1989a; van Deuren et al., 2000; Brandtzaeg et al., 2001; Ovstebo et al., 2004; Brandtzaeg, 2006; Stephens et al., 2007; Brandtzaeg and van Deuren, 2012; Brusletto et al., 2017; Brusletto et al., 2020). Autopsy studies of lungs, heart, kidneys, liver, and spleen of patients with meningococcal septic shock indicate that the concentrations of *N. meningitidis* are present in all major organs, varying from 10^6^ to 10^8^ copy numbers of *N. meningitidis* DNA per microgram of human DNA (Brusletto et al., 2017; Brusletto et al., 2020). The consequence for the patients is a rapid and detrimental activation of major arms of the immune system (Brandtzaeg, 1995; de Kleijn et al., 1998; van Deuren et al., 2000; Brandtzaeg et al., 2001; Stephens et al., 2007; Brandtzaeg and van Deuren, 2012; Hellerud et al., 2015; Brusletto et al., 2020). This activation includes release of pro- and anti-inflammatory cytokines in plasma and tissues, high graded activation of the complement system generating high levels of complement factors C3a, C4a, and C5a, release of vasoactive intestinal peptide (VIP) from the gastrointestinal tract, and gradually increasing disseminated intravascular coagulation (DIC) induced by activation of circulating tissue factor (TF) on monocytes and microparticles derived from monocytes (Osterud and Flaegstad, 1983; Brandtzaeg et al., 1989a; Brandtzaeg et al., 1989b; Brandtzaeg et al., 1989c; Waage et al., 1989; Brandtzaeg, 1995; Hazelzet et al., 1996; Kornelisse et al., 1996; de Kleijn et al., 1998; van Deuren et al., 2000; Brandtzaeg et al., 2001; Rosenstein et al., 2001; Brandtzaeg, 2006; Brandtzaeg and van Deuren, 2012; Hellum et al., 2014). The levels of TF on the microparticles are closely associated with plasma levels of LPS (Hellum et al., 2014). Concomitantly, plasminogen activator inhibitor 1 (PAI-1) exposed on the vessel walls is released into the circulation and thereby inhibits the fibrinolytic system (Brandtzaeg et al., 1990; Kornelisse et al., 1996; Brusletto et al., 2020).

The clinical picture of meningococcal septic shock is characterized by a short time between the recognition of the first disease symptoms to hospital admission (van Deuren et al., 2000; de Greeff et al., 2008; Brandtzaeg and van Deuren, 2012). Studies from the Netherlands and Norway suggest that the median onset – admission time during the serogroup B and C meningococcal epidemics, was 12 h for shock patients compared with 26 h for patients with distinct symptoms of meningitis without shock (van Deuren et al., 2000; de Greeff et al., 2008; Brandtzaeg and van Deuren, 2012). The patients become febrile, and complain of malaise, muscle pains, nausea, diarrhea, cold extremities, and gradually increasing skin hemorrhages, particularly on the extremities. The circulation is deteriorating to septic shock accompanied with increasing pulmonary and renal failure. Fifty percent of the patients die within 12 h of hospital admission and death is correlated to the admission levels of lipopolysaccharides (LPS) in plasma (Brandtzaeg et al., 1989a; Brandtzaeg et al., 1989d; van Deuren et al., 2000; Brandtzaeg et al., 2001; Stephens et al., 2007; Brandtzaeg and van Deuren, 2012).

The underlying pathophysiology of meningococcal septic shock and multiple organ failure has primarily been established by analysis of autopsy material and more recently by blood and tissue samples. Comparatively, little new information has emerged from autopsies studies since the major articles were published in the 1940s (Martland, 1944; D'Agati and Marangoni, 1945; Wright and Reppert, 1946; Ferguson and Chapman, 1948). However, this has changed lately. By using transcriptomic methods to evaluate the changes in gene expression in human immune cells, *in vitro* experiments, and tissues from lungs, heart, kidneys, liver, and spleen of deceased meningococcal shock patients, the biological complexity involving thousands of genes has become obvious (Ovstebo et al., 2004; Ovstebø et al., 2008; Gopinathan et al., 2012; Gopinathan et al., 2015; Hellerud et al., 2015; Brusletto et al., 2020). The results suggest that human immune cells in the lungs, heart, kidneys, liver, and spleen reveal a comparatively specific transcriptomic pattern as a reaction to the massive insult of intruding *N. meningitidis* (Gopinathan et al., 2015; Brusletto et al., 2020).

To better understand the pathological changes observed in the patients, we have developed a porcine model simulating fulminant human meningococcal sepsis and multiple organ failure (Nielsen et al., 2009; Hellerud et al., 2015; Hellerud et al., 2017), which reflects the prehospital and early hospital stages. By infusing escalating doses of the epidemic reference strain of *N. meningitidis* (H44/76) intravenously and comparing the results with a mutant meningococcal strain completely lacking LPS in the outer membrane (H44/76*lpxA-*), the potent role of LPS in meningococcal septic shock and multiple organ failure has been firmly established (Holten, 1979; Steeghs et al., 1998; Nielsen et al., 2009; Hellerud et al., 2010). The model also documented the inflammation-inducing capacity of non-LPS molecules of *N. meningitidis*, which are weaker, but still potent in high concentrations (Hellerud et al., 2008; Hellerud et al., 2010). The porcine model complemented the picture of the released key molecules related to the rapidly escalating and devastating inflammation, coagulation, and fibrinolysis and the altered regulation of the genes coding these molecules.

In this study, we have compared the changes in the human transcriptomes obtained from five meningococcal patients with lethal septic shock and multiple organ failure with the transcriptomes from the porcine model of meningococcal shock and multiple organ failure. The tissues from the patients were obtained at the routine autopsy within 24 h of death and kept as formalin-fixed, paraffin-embedded (FFPE) material at room temperature (+20°C) (Brusletto et al., 2020). In addition, tissues from the same organs from three patients were fresh frozen and kept at −80°C, and the transcriptomic changes were compared to the FFPE material (Brusletto et al., 2020). The organ materials from the porcine model were fresh frozen at −80°C immediately after the autopsy (Hellerud et al., 2015).

We asked a key question: how well are the transcriptomic changes in the patient material reflected in the porcine model? Can a porcine model be used to test new experimental therapies, which is difficult to evaluate in meningococcal patients due to the rarity of the disease in industrialized countries that makes randomized clinical controlled trials very difficult to perform? A newly conducted experiment on a porcine model on complement inhibition and inflammation suggests that we may obtain valuable information about possible intervention strategies (Hellerud et al., 2017). Large epidemics of serogroups A, B, C, W, and Y will presumably rarely or never occur in the economically developed world in the future since highly protective vaccines are presently available for the five major serogroups and a vaccine for serogroup X is under development (Dretler et al., 2018; Oldrini et al., 2018). These vaccines make long-lasting epidemics unlikely and alternative ways for testing new treatment strategies should be sought. A further developed porcine model could be one alternative reflecting human gene reaction patterns.

## Materials and methods

### Animals used in the experiments

Norwegian landrace pigs of both sexes with a bodyweight of 30 kg were used (Nielsen et al., 2009; Hellerud et al., 2015; Hellerud et al., 2017).

### Bacterial reference strain

*Neisseria meningitidis* (44/76, B;15:P1,16:L3,7,9 also known internationally as H44/76) causing epidemics in North-West Europe from 1975 was used (Holten, 1979; Bjune et al., 1991; Piet et al., 2011).

### Quantification of *Neisseria meningitidis* (Nm) DNA in formalin-fixed, paraffin-embedded (FFPE) and fresh frozen (FF) tissue from patients with meningococcal shock and multiple organ failure and from FF tissue samples from a porcine experimental model

The bacterial load of *N. meningitidis* DNA was analyzed using quantitative real-time PCR (q-PCR) and primers for the capsule transport A (ctrA) gene (Frosch et al., 1992) (1 copy per *N. meningitidis* DNA) (Hellerud et al., 2015; Brusletto et al., 2017). In the human model, the results were depicted as DNA copies/μg human DNA, and in the porcine model, the results were depicted as DNA copies/g tissue.

### Comparison of transcription profiles in the human patients Nm septic shock model and the porcine Nm septic shock experimental model

Gene expression data from two different, previously published models of transcriptomic changes induced in a porcine model infused with the *N. meningitidis* reference strain H44/76 and a human meningococcal septic shock autopsy study were used for this study (Hellerud et al., 2015; Brusletto et al., 2020) (**Table 1**). The data and protocols for both studies are compliant with the minimum information about microarray experiment (MIAME) guidelines (Brazma et al., 2001).

**Table 1:** Experimental conditions for DNA microarray data.

Model	Experimental conditions		Type of storage methods	Type of organ tissue	Type of array	Array description
Human patients Nm septic shock model	Patients (*n* = 5) with meningococcal septic shock (MSS)	Controls (*n* = 2) (Patients with non-infectious disease, sudden death)	FFPE (formalin-fixed, paraffin-embedded)	Lungs, Heart, Kidneys, Liver, and Spleen	Affymetrix GeneChip Human Transcriptome 2.0 Arrays	The array contains 44,699 protein-coding genes and 22,829 non-protein-coding genes
Porcine Nm septic shock experimental model	Pigs (*n* = 8) received exponentially increasing numbers of *N. meningitidis*	Control pigs (*n* = 3) received 0.9% NaCl	FF (fresh frozen tissue)	Lungs, Kidneys, Liver, and Spleen	Affymetrix GeneChip Porcine Genome Array	The array contains 23,937 probe sets that interrogate approximately 23,256 transcripts from 20,201 *S. scrofa* genes

In the porcine model (Hellerud et al., 2015), the gene expression changes in tissues from pigs (*n* = 8), which received exponentially increasing numbers of *N. meningitidis* (reference strain H44/76) during 4 h with a duplication time of 30 min, were analyzed. The control group was three pigs receiving 0.9% NaCl only. No animals died during the experiment. At the end of the experiments, the animals were euthanized and biopsies were obtained from the lungs, liver, spleen, and kidneys and rapidly frozen on liquid nitrogen. In the human model (Brusletto et al., 2020), FFPE (*n* = 5) or fresh frozen (FF) (*n* = 3) tissues from lungs, heart, kidneys, liver, and spleen from patients with meningococcal shock and multiple organ failure were collected after the routine autopsy examination within 24 h after the patient died, stored at RT or −80°C, respectively, and analyzed for gene expression changes (Brusletto et al., 2020). FFPE tissues from two patients with a non-inflammatory disease were used as negative controls (Brusletto et al., 2020).

In this study, we compared gene expression data from two different studies. The types of microarrays used were different. In the human meningococcal septic shock study, the array contains protein-coding genes as well as non-protein-coding genes; however, for the porcine experimental model, the array contains only protein-coding genes (**Table 1**).

### Ingenuity pathway analysis

Data from previously generated gene expression profiles induced in the porcine model (Hellerud et al., 2015) and in the patient model (Brusletto et al., 2020) were imported into the Ingenuity pathway analysis for core and comparison analysis in 2021 and resulted in additional activated pathways than previously published, due to a continuous updating of IPA.

Gene lists (Excel files) containing gene identifiers (probe set IDs), and corresponding *p*-values were uploaded to Ingenuity Pathway Analysis (IPA, Ingenuity Systems, www.ingenuity.com). A cutoff at FDR < 5% was set to identify significantly differentially expressed genes. In order to compare the similarities and differences among the enriched pathways and upstream regulators, the comparison analysis function in IPA was used. The canonical pathway tool was used to identify the top canonical pathways associated with the genes differentially expressed between the compared conditions. Biological functions associated with the differentially expressed genes were identified by mapping each gene to its corresponding function in the Ingenuity Knowledge Base. To identify potential triggers of the differential gene expression, the data were analyzed to find upstream regulators that drive similar gene expression profiles. The right-tailed Fisher’s exact test was used to calculate a *p*-value determining the probability that each canonical pathway, biological function, and upstream regulator assigned to the dataset was due to chance alone. The *p*-values were corrected for multiple comparisons using the Benjamini–Hochberg method for correcting the FDR. The results were expressed as fold changes (FCs). Transcripts with FC ≥ | ± 2| and *p*-values < 0.05 were regarded as significantly regulated.

Enrichment analyses were performed using IPA’s ≪core analysis≫ for each tissue sample. These analyses have the ability to identify significantly activated biological functions and pathways, molecular functions, and relationships in our dataset of genes. A right-tailed Fisher’s exact test calculated *p*-values corrected for multiple testing by the Benjamini-Hochberg method. In addition, an “Upstream regulator” analysis was used to detect the cascade of upstream transcriptional regulators that were involved in the porcine Nm septic shock model and in the human Nm septic shock patients, and whether they were likely activated or inhibited to acquire the observed gene expression profile changes in our datasets.

IPA’s Z-score indicates a predicted activation or inhibition of canonical pathways, genes, biofunctions, and upstream regulators, which are assigned as inhibited or activated according to Z-score value >| ± 2|. An absolute Z-score of ≥2 is considered significant. IPA does not assign predictions to any values between 2 and −2.

## Results

### Quantification of *Neisseria meningitidis* DNA in FFPE and FF tissue from patients with meningococcal shock and multiple organ failure and from FF tissue samples from a porcine experimental model

*N. meningitidis* DNA was detected in all FFPE tissues from patients with severe shock and multiple organ failure (Brusletto et al., 2017) and is shown in **Table 2**. The median concentrations of *N. meningitidis* DNA in the organs (FFPE tissue) ranged from 9.1 × 10e4 to 2.4 × 10e7 copies of *N. meningitidis* DNA/μg human DNA (**Table 2**). The median concentrations of *N. meningitidis* DNA in the FF tissue samples from patients with severe shock and multiple organ failure ranged from 2.5 × 10e7 to 2.3 × 10e8 copies of *N. meningitidis* DNA/μg human DNA (**Table 2**).

**Table 2:** Quantification of *N.meningitidis* (Nm) DNA in human and porcine tissue samples by q-PCR. In humans, Nm DNA presented as median numbers of *Nm* DNA copies/μg human DNA and in porcinis as median numbers of DNA copies/g tissue.

Model	Type of organ tissue	Median numbers Nm DNA in FFPE	Type of organ tissue	Median numbers Nm DNA in FF
		**DNA copies /μg human DNA**		**DNA copies /μg human DNA**
**Human patients Nm septic shock model**	Lungs (n=5)	1.5x10e7	Lungs (n=3)	2.3x10e8
	Heart (n=4)	2.4 x10e7	Heart (n=3)	3.6x10e7
	Kidneys (n=4)	2.0x10e6	Kidneys (n=3)	6.3x10e7
	Liver (n=4)	7.0x10e5	Liver (n=2)	7.0x10e7
	Spleen (n=3)	9.1x10e4	Spleen (n=2)	2.5x10e7
				**DNA copies /g tissue**
**Porcine Nm septic shock experimental model**			Lungs (n=8)	1.0x10e6/g
			Kidneys (n=8)	Below detection limit (1x10e4 bacteria/g tissue)
			Liver (n=8)	8.0x10e5/g
			Spleen (n=8)	Below detection limit (1x10e4 bacteria/g tissue)

Median number of *N. meningitidis* DNA copies from FF tissue samples in the porcine organs was 1 × 10e6/g tissue in the lungs, 8 × 10e5/g tissue in the liver and below detection limit in the rest of the organs (**Table 2**) (Hellerud et al., 2015).

### Gene expression profiles in formalin-fixed, paraffin-embedded tissue samples from meningococcal septic shock patients

#### Descriptive analysis and predicted biological functions of differentially expressed genes in FFPE tissue samples from meningococcal septic shock patients

An updated IPA ≪core analysis≫ was performed separately for each organ to fine-tune the top enriched pathways (**Additional file 1_Figure S1**). Each organ displays different activated canonical pathways. In the lungs, heart, and kidneys, many similar pro-inflammatory pathways are activated, such as Acute Phase Response Signaling. The EIF2 Signaling pathway and the PPAR Signaling pathway, however, are in different degrees downregulated in heart and kidneys. In lungs and heart, the canonical pathways IL-17, TREM, IL-6, and HMBG1 Signaling and activation of the pathway Differential regulation of cytokine production in macrophages and T-helper cells by IL-17A and IL-17F are found upregulated (**Additional file 1_Figure S1**). Upstream regulators (**Table 3**), such as pro-inflammatory regulators, are highly activated in lungs, while transcription factors and translation regulators that regulate signaling pathways involved in development, growth, repair, and homeostasis, and triglyceride synthesis are found highly activated in other organs. This new core analysis describes a more complex immune activation with high involvement of the adaptive immune system. In liver and spleen, few canonical pathways are activated.

**Table 3:** Top upstream regulators differentially expressed (A) organs from five patients with meningococcal septic shock ^a^ versus control (B) organs from eight porcinis infused exponentially with increasing numbers of *N. meningitidis* (reference strain H44/76) ^b^ vs. controls.

A	Human Lungs	Human Heart	Human Kidneys	Human Liver	Human Spleen
**Top Upstream Regulators** *p*-value of overlap and predicted activation ^a^	TNF 5.68E-26 (Activated)	LARP1 5.04E-48 (Inhibited)	RICTOR 9.97E-17 (Inhibited)	RXRA 2,65E-11	ATF4 1.23E-04
	IL1A 9.33E-22 (Activated)	MYC 2.18-38 (Activated)	LARP1 5.03E-15 (Inhibited)	NR1I2 6.58E-09	SBDS 1.99E-04
	IL1B 2.78E-20	MYCN 4.42E-37 (Activated)	MLXIPL 2.91E-14 (Activated)	ACOX1 7.65E-09	CREB1 6.93E-04
	NFkB (complex) 1.22E-19 (Activated)	YAP1 4.51E-32	YAP1 2.28E-12	PPARA 3.55E-08	CAB39L 1.11E-03
	TGFB1 2.40E-19 (Activated)	MLXPL 7.06E-29 (Activated)	MYC 2.65E-12 (Activated)	APP 2.51 E-07	TFAP2D 1.29-03
**B**	**Porcine Lungs**		**Porcine Kidneys**	**Porcine Liver**	**Porcine Spleen**
**Top Upstream Regulators** *p*-value of overlap and predicted activation ^b^	IL1B 7.41E-47 (Activated)		IL1B 2.57E-43 (Activated)	IL1B 8.03E-48 (Activated)	IL1B 2.31E-37 (Activated)
	TGFB1 9.95E-45 (Activated)		TNF 5.15E-43 (Activated)	TNF 1.11E-47 (Activated)	IL6 5.42E-32 (Activated)
	TNF 1.90E-38 (Activated)		PPARA 2.07E-40	IFNG 2.14E-41 (Activated	TNF 2.15E-31 (Activated
	NFkB (complex)1.19E-37(Activated)		IL6 1.01E-39 (Activated)	IL-6 9.88E-39 (Activated)	NR3C1 3.84E-29 (Inhibited)

a Functional “core” analysis performed by IPA. p-values of overlap comparing values from meningococcal septic shock (n = 5) patients with controls (n = 2) (filtering criteria: FC ≥| ± 2|, p < 0.05).

b Functional “core” analysis performed by IPA. p-values of overlap comparing values from porcinis (n = 8) infused exponentially increasing numbers of *N. meningitidis* (reference strain H44/76) vs. controls (n = 3) (filtering criteria: FC ≥| ± 2|, p < 0.05).

### Gene expression profiles in fresh frozen tissue samples from the porcine Nm septic shock experimental model

#### Descriptive analysis and predicted biological functions of differentially expressed genes in FF tissue samples from the porcine Nm septic shock experimental model

The renewed May 2021 version IPA ≪core analysis ≫ of the porcine tissues showed that each organ displayed different upregulated activated canonical pathways, such as HMBG1 Signaling, Role of Pattern Recognition Receptors in Recognition of Bacteria and Viruses, TREM 1 Signaling, IL-17 Signaling, IL-6 Signaling, Crosstalk between Dendritic Cells and Natural Killer Cells, and Acute Phase Response Signaling (**Additional file 2_Figure S2**). The main downregulated pathways in all the organs were LXR/RXR Activation pathway as well as Erythropoietin Signaling pathway in kidneys and spleen (**Additional file 2_Figure S2**). The analysis of upstream regulators showed IL1B, TNF, IFNG, and NFκB on top (**Table 3**).

### Comparison analysis: Predicted biological functions of differentially expressed genes in tissue samples from meningococcal septic shock patients versus the porcine Nm septic shock experimental model

**Figure 1:**
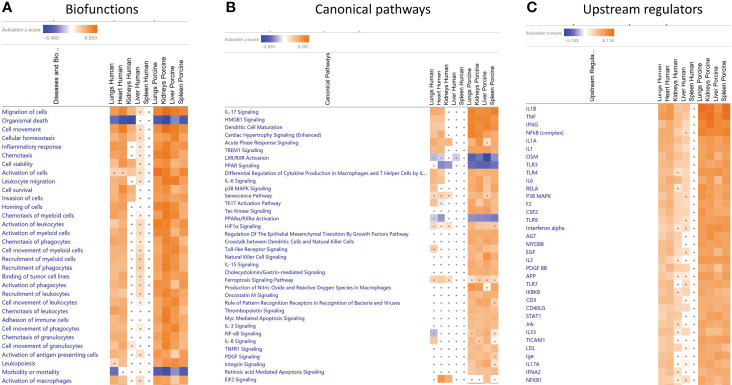
Predicted biofunctions **(A)**, canonical pathways **(B)**, and upstream regulators **(C)** in FFPE tissue samples from patients with meningococcal septic shock and in organs from porcinis infused with exponentially increasing numbers of *N. meningitidis* (reference strain H44/76) vs. controls. ≪Comparison analysis≫ of biofunctions **(A)**, canonical pathways **(B)**, and upstream regulators **(C)**, significantly enriched in FFPE tissue samples from lungs, heart, kidneys, liver, and spleen from meningococcal septic shock patients vs. control patients (acute non-infectious death) and in FF tissue samples from lungs, kidneys, liver and spleen from porcinis infused with exponentially increasing numbers of *N. meningitidis* (reference strain H44/76) vs. controls was performed. The figure shows the most upregulated biofunctions **(A)**, canonical pathways **(B)**, and upstream regulators **(C)** ranked according to expression levels in FFPE tissues from lungs. The Z-score indicates predicted activation state of the biofunctions, canonical pathways, and upstream regulators. Z-score values >| ± 2| are displayed. Dots display insignificant threshold Z-score values <± 2|. Blue color or lighter shades of blue indicate a negative Z-score and downregulation of a biofunction, canonical pathways, and upstream regulators. Orange or lighter shades of orange indicate a positive Z-score and upregulation of a biofunction, canonical pathways, and upstream regulators. Note that only the top pathways are shown.

When gene expression changes in the tissue samples from meningococcal septic shock patients were compared with gene expression changes in the porcine experimental model, the IPA “comparison analysis” (**Figure 1**) predicted the downregulated biofunctions (**Figure 1A**) to be organismal death and mortality. The top upregulated biofunctions were predicted to be migration of cells, cell movement, cellular homeostasis, inflammatory response, chemotaxis, cell viability, activation of cells, leukocyte migration, cell survival, invasion of cells, and horning of cells (**Figure 1A** and **Table 4**). The main differences between the two experiments seem to be that the involvement, activation, and movement of blood cell, leukocytes, and myeloid cells are more upregulated in the porcine experimental model than in the human organs.

**Table 4:** Predicted biofunctions based on Z-score values in FFPE tissue samples from patients with meningococcal septic shock and in organs from porcinis infused with exponentially increasing numbers of *N. meningitidis* (reference strain H44/76) vs. controls.

	Z-score	Z-score	Z-score	Z-score	Z-score	Z-score	Z-score	Z-score	Z-score
Diseases and Bio Functions	Lungs Human	Heart Human	Kidneys Human	Liver Human	Spleen Human	Lungs Porcine	Kidneys Porcine	Liver Porcine	Spleen Porcine
Migration of cells	4.0	4.9	3.0	1.2	N/A	4.9	6.0	5.3	4.8
Organismal death	−3.7	−5.4	−6.5	N/A	N/A	−4.8	−6.0	−3.0	−4.8
Cell movement	4.0	5.1	3.2	1.4	N/A	4.6	6.0	5.1	4.5
Cellular homeostasis	3.1	3.9	2.7	1.6	N/A	3.9	4.1	5.0	3.6
Inflammatory response	4.2	4.0	N/A	1.0	N/A	4.4	5.2	4.1	4.3
Chemotaxis	3.7	3.9	N/A	1.0	N/A	5.1	5.4	4.3	3.4
Cell viability	2.6	4.3	3.3	1.0	N/A	4.3	4.6	3.0	3.6
Activation of cells	1.8	1.8	2.3	1.4	N/A	5.8	5.1	3.7	4.8
Leukocyte migration	3.1	3.4	N/A	1.2	N/A	4.3	5.4	5.2	3.9
Cell survival	3.0	4.6	3.1	N/A	N/A	4.2	4.6	2.8	3.4
Invasion of cells	3.1	3.8	2.7	N/A	N/A	4.0	4.2	3.7	4.1
Homing of cells	3.7	3.9	N/A	N/A	N/A	5.0	5.3	4.5	3.3
Chemotaxis of myeloid cells	3.9	3.4	N/A	0.7	N/A	4.0	5.3	4.7	3.2
Activation of leukocytes	2.2	3.1	N/A	1.5	N/A	5.3	5.0	3.6	4.5
Activation of myeloid cells	3.1	3.5	N/A	1.4	N/A	4.8	4.8	2.9	4.1
Chemotaxis of phagocytes	3.7	3.4	N/A	0.9	N/A	3.7	5.0	4.5	2.8
Cell movement of myeloid cells	3.1	3.2	N/A	1.3	N/A	3.4	4.6	4.8	3.5
Recruitment of myeloid cells	3.0	2.9	N/A	2.0	N/A	3.8	4.9	4.3	3.0
Recruitment of phagocytes	2.8	2.8	N/A	1.9	N/A	3.8	5.1	4.3	3.0
Binding of tumor cell lines	2.7	3.6	N/A	1.5	N/A	3.5	4.5	3.8	4.1
Activation of phagocytes	3.1	3.4	N/A	1.4	N/A	4.5	4.4	2.4	4.2
Recruitment of leukocytes	2.7	2.3	N/A	1.4	N/A	4.2	4.9	4.2	3.6
Cell movement of leukocytes	3.1	3.3	N/A	0.7	N/A	3.5	4.4	4.9	3.2
Chemotaxis of leukocytes	3.9	3.6	N/A	N/A	N/A	3.6	5.0	4.1	2.6
Adhesion of immune cells	2.8	3.3	N/A	N/A	N/A	4.0	5.2	4.0	3.4
Cell movement of phagocytes	2.9	2.8	N/A	0.7	N/A	3.3	4.5	4.9	3.3
Chemotaxis of granulocytes	3.2	3.3	N/A	N/A	N/A	3.9	4.8	4.1	2.9
Cell movement of granulocytes	2.5	3.6	1.0	N/A	N/A	3.4	4.9	3.9	2.9
Activation of antigen presenting cells	3.2	2.8	N/A	1.2	N/A	4.2	3.7	2.0	4.0
Leukopoiesis	1.6	2.7	N/A	0.0	N/A	3.6	4.5	4.6	4.2
Morbidity or mortality	−3.6	N/A	N/A	N/A	N/A	−4.6	−5.5	−2.8	−4.6
Activation of macrophages	3.4	2.6	N/A	1.4	N/A	4.0	3.7	2.2	3.7

≪Comparison analysis≫ of biofunctions significantly enriched in FFPE tissue samples from lungs, heart, kidneys, liver, and spleen from meningococcal septic shock patients vs. control patients (acute non-infectious death) and in FF tissue samples from lungs, kidneys, liver, and spleen from porcinis infused with exponentially increasing numbers of *N. meningitidis* (reference strain H44/76) vs. controls were performed. The table shows the top most upregulated biofunctions ranked according to expression levels in FFPE tissues from lungs. The Z-score indicates predicted activation state of the biofunctions. Note that only the top biofunctions are shown. N/A = not applicable.

The main upregulated canonical pathways (**Figure 1B** and **Table 5**) were predicted to be the IL-17 signaling, HMBG1 signaling, Dendritic Cell Maturation, Cardiac Hypertrophy signaling, Acute phase Response Signaling, and TREM signaling whereas LXR/RXR Activation, PPAR signaling, and PPARα/RXRα Activation were predicted to be the most downregulated. The main differences in canonical pathways between the two types of experiments were the different expressions of NF-κB. NF-κB signaling was downregulated in the human lung tissue samples, whereas an upregulated activation was observed in the porcine organs. In addition, the Tec Kinase Signaling pathway was differentially regulated, absent in the human model and highly expressed in the porcine model (**Figure 1B** and **Table 5**). The EIF2 signaling pathway that is highly upregulated in heart and kidneys in the human model is also present and upregulated in the porcine liver.

**Table 5:** Predicted canonical pathways based on Z-score values in FFPE tissue samples from patients with meningococcal septic shock and in organs from porcinis infused with exponentially increasing numbers of *N. meningitidis* (reference strain H44/76) vs. controls.

	Z-score	Z-score	Z-score	Z-score	Z-score	Z-score	Z-score	Z-score	Z-score
Canonical Pathways	Lungs Human	Heart Human	Kidneys Human	Liver Human	Spleen Human	Lungs Porcine	Kidneys Porcine	Liver Porcine	Spleen Porcine
IL-17 Signaling	3.0	2.8	N/A	N/A	N/A	4.4	4.0	4.1	4.5
HMGB1 Signaling	2.2	2.2	N/A	N/A	N/A	4.2	4.2	4.2	3.8
Dendritic Cell Maturation	2.0	2.0	N/A	N/A	N/A	4.4	4.0	4.4	3.9
Cardiac Hypertrophy Signaling (Enhanced)	2.2	3.0	N/A	N/A	N/A	4.4	4.1	3.3	3.0
Acute Phase Response Signaling	3.0	2.4	2.6	N/A	N/A	2.7	3.9	1.1	3.0
TREM1 Signaling	2.4	2.6	N/A	N/A	N/A	3.4	3.3	3.4	2.8
LXR/RXR Activation	−1.3	−1.0	N/A	−1.0	N/A	−3.3	−2.7	−5.0	−2.1
PPAR Signaling	N/A	−2.2	−2.0	N/A	N/A	−2.8	−3.2	−3.2	−2.7
Differential Regulation of Cytokine Production in Macrophages and T Helper Cells by IL-17A and IL-17F	2.6	2.0	N/A	N/A	N/A	3.0	2.6	2.8	2.8
IL-6 Signaling	2.4	2.2	N/A	N/A	N/A	2.8	3.4	2.2	2.8
p38 MAPK Signaling	2.2	N/A	N/A	N/A	N/A	3.0	3.5	2.1	3.3
Senescence Pathway	1.3	1.6	1.3	N/A	N/A	2.5	2.7	1.7	2.5
Th17 Activation Pathway	2.2	−0.4	N/A	N/A	N/A	3.2	2.0	3.0	2.3
Tec Kinase Signaling	N/A	N/A	N/A	N/A	N/A	3.3	3.2	3.5	2.5
PPARα/RXRα Activation	−1.0	−2.0	N/A	N/A	N/A	−2.1	−2.2	−2.3	−2.5
HIF1α Signaling	1.3	0.7	1.1	N/A	N/A	2.7	2.1	2.0	1.9
Regulation of the Epithelial Mesenchymal Transition by Growth Factors Pathway	N/A	N/A	N/A	N/A	N/A	3.0	3.0	2.9	3.1
Crosstalk between Dendritic Cells and Natural Killer Cells	N/A	N/A	N/A	N/A	N/A	3.1	2.7	3.2	2.9
Toll-like Receptor Signaling	1.9	N/A	N/A	N/A	N/A	2.6	2.8	2.3	2.1
Natural Killer Cell Signaling	N/A	N/A	N/A	N/A	N/A	2.6	2.2	3.9	2.9
IL-15 Signaling	N/A	N/A	N/A	N/A	N/A	3.0	2.6	3.2	2.5
Cholecystokinin/Gastrin-mediated Signaling	N/A	N/A	N/A	N/A	N/A	2.8	2.8	2.8	2.6
Ferroptosis Signaling Pathway	1.6	2.0	1.3	N/A	N/A	1.1	1.9	1.7	1.3
Production of Nitric Oxide and Reactive Oxygen Species in Macrophages	N/A	N/A	N/A	N/A	N/A	3.7	3.5	0.4	3.2
Oncostatin M Signaling	N/A	N/A	N/A	N/A	N/A	2.6	2.4	2.8	2.8
Role of Pattern Recognition Receptors in Recognition of Bacteria and Viruses	N/A	N/A	N/A	N/A	N/A	2.5	3.2	3.2	1.7
Thrombopoietin Signaling	N/A	N/A	N/A	N/A	N/A	2.6	2.4	2.6	2.3
Myc Mediated Apoptosis Signaling	N/A	N/A	N/A	N/A	N/A	2.4	2.3	2.4	2.6
IL-3 Signaling	N/A	N/A	N/A	N/A	N/A	2.4	2.2	2.6	2.3
NF-κB Signaling	−1.3	N/A	N/A	N/A	N/A	1.9	2.5	2.8	1.0
IL-8 Signaling	2.0	N/A	N/A	N/A	N/A	2.5	1.9	2.7	0.3
TNFR1 Signaling	N/A	N/A	N/A	N/A	N/A	2.2	2.4	2.0	2.4
PDGF Signaling	N/A	N/A	N/A	N/A	N/A	2.4	2.2	2.4	1.9
Integrin Signaling	N/A	N/A	N/A	N/A	N/A	2.0	2.6	3.2	1.1
Retinoic acid Mediated Apoptosis Signaling	N/A	N/A	N/A	N/A	N/A	2.0	2.2	2.2	2.4
EIF2 Signaling	N/A	3.8	2.8	N/A	N/A	N/A	N/A	2.2	N/A

≪Comparison analysis≫ of canonical pathways significantly enriched in FFPE tissue samples from lungs, heart, kidneys, liver, and spleen from meningococcal septic shock patients vs. controls patients (acute non-infectious death) and in FF tissue samples from lungs, kidneys, liver, and spleen from porcinis infused with exponentially increasing numbers of *N. meningitidis* (reference strain H44/76) vs. controls were performed. The table shows the top most upregulated canonical pathways ranked according to expression levels in FFPE tissues from lungs. The Z-score indicates predicted activation state of the canonical pathways. Note that only the top canonical pathways are shown. N/A = not applicable.

**Figure 2:**
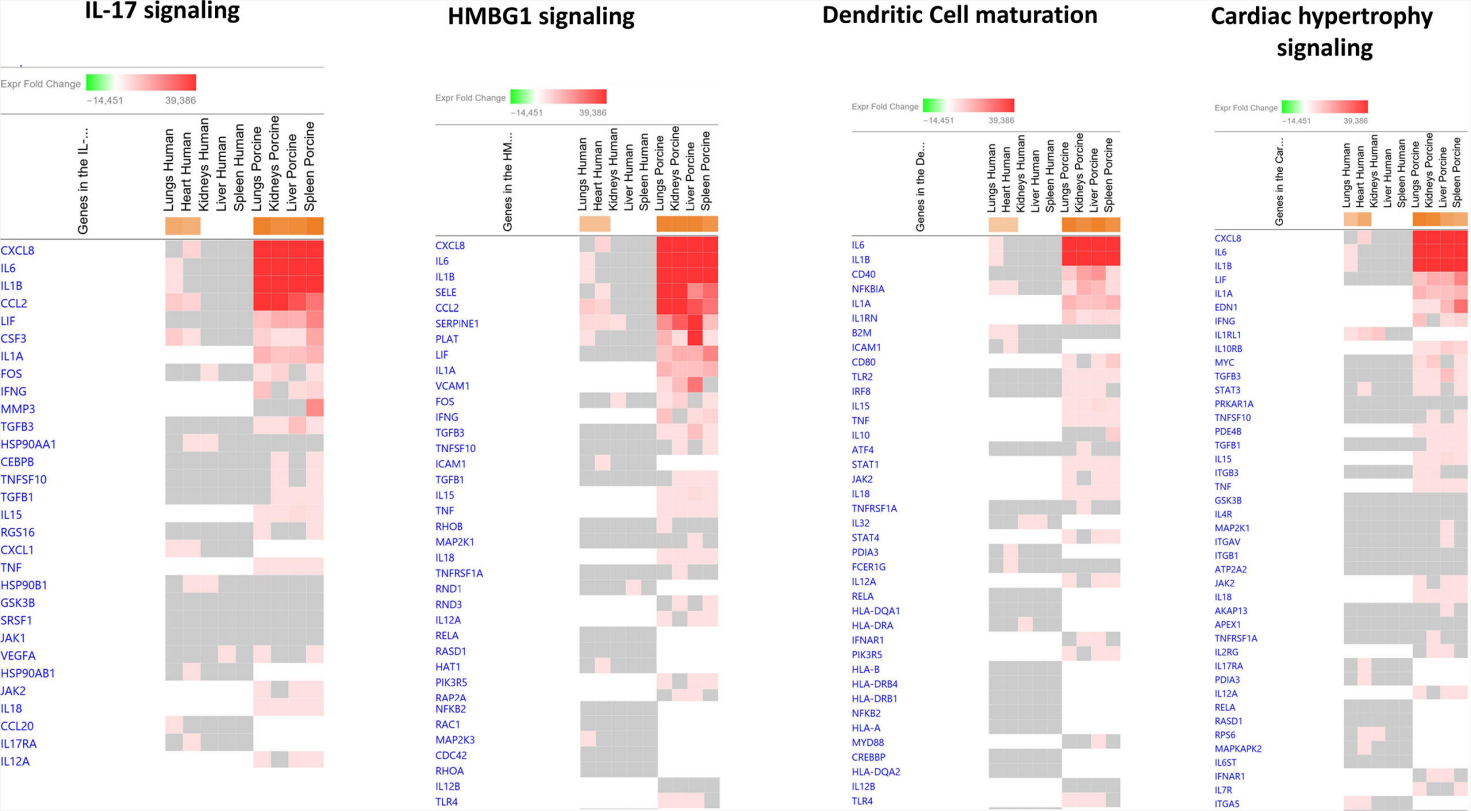
Predicted gene signaling pathways from the top upregulated canonical pathways in FFPE tissue samples from patients with meningococcal septic shock and in organs from porcinis infused with exponentially increasing numbers of *N. meningitidis* (reference strain H44/76) vs. controls. The Z-score indicates predicted activation state of canonical pathways in the tissue samples from meningococcal septic shock patients vs. controls and in organs from porcinis infused with exponentially increasing numbers of *N. meningitidis* (reference strain H44/76) vs. controls. Orange or lighter shades of orange indicate a positive Z-score and upregulation of the pathway. The transcripts in the gene signaling pathway are expressed as fold change (FC) values. Red or lighter shades of red indicate positive FC-values and upregulation of transcripts; green color or lighter shades of green indicate negative FC-values and downregulation of transcripts. The color gray indicates that a predicted activation state of a gene/transcript in the canonical pathway signaling network is not affected, the gene/transcript was in the dataset but did not pass the analysis cutoffs. White color indicates that gene/transcript are not present in the dataset.

Among the predicted top upregulated canonical pathways, the genes CXCL8, IL1B, CCL2, SERPINE1, NFKB1A, and IL-6 were on top (**Figure 2** and **Additional File 3 _Table 1**). CXCL8 was on top in IL-17 signaling, HMBG1 signaling, and Cardiac Hypertrophy signaling while IL-6 was on top in Dendritic Cell maturation. In the human study, CXCL8 was significantly upregulated only in heart tissue and IL-6 was significantly upregulated in lung tissue. Different expression patterns were also found in the two models when exploring predicted upregulated genes. IL1RL1, ICAM1, CXCL1, ITGA5, and IL32 were only present and upregulated in the human organs. Upregulated genes unique for the porcine model were IL1A, IFNG, VCAM1, IL1RN, TNF, IL-10, EDN1, and IL-18 (**Figure 2** and **Additional File 3_Table 1**).

**Figure 3:**
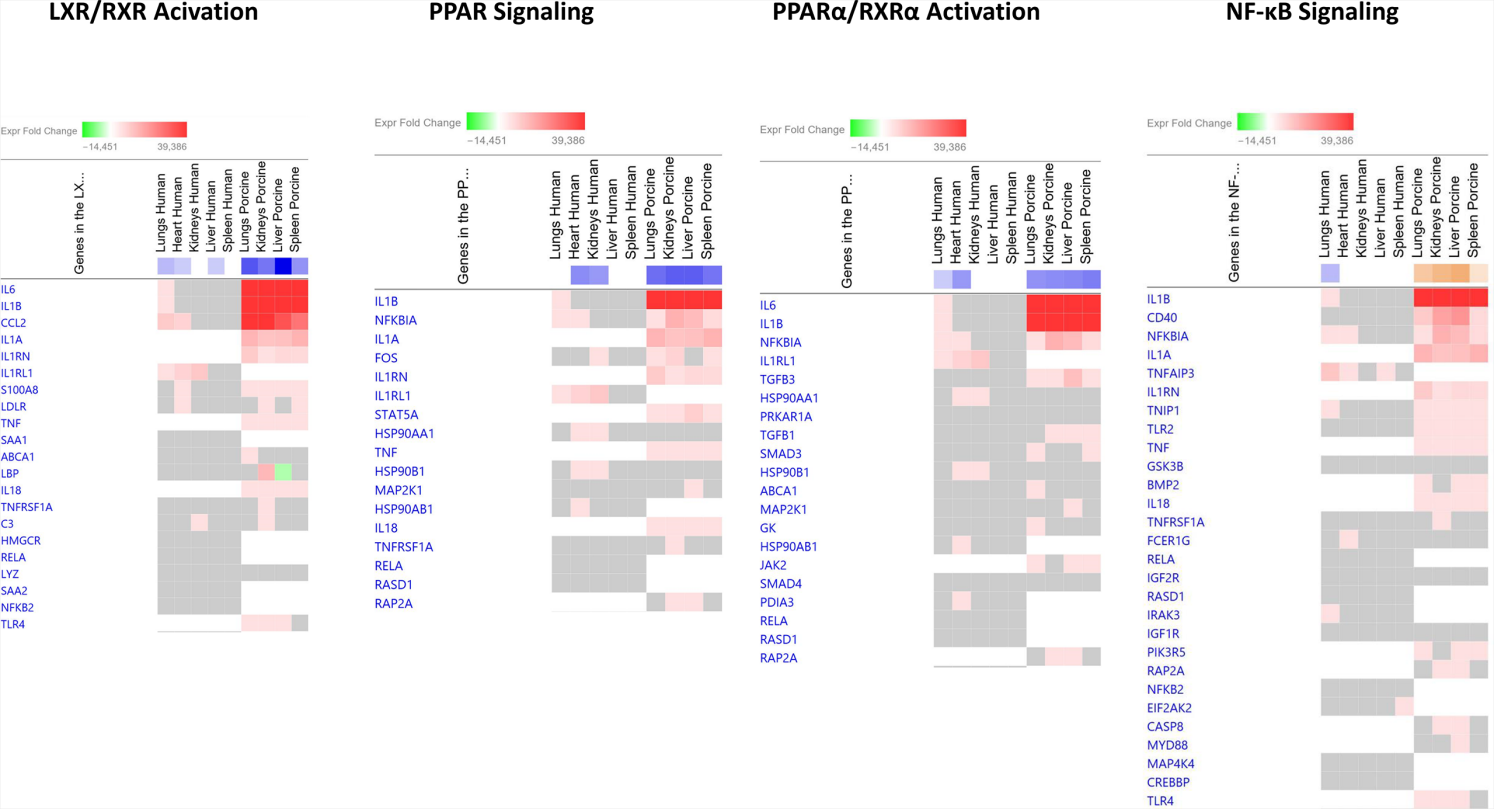
Predicted gene signaling pathways from the top downregulated canonical pathways in FFPE tissue samples from patients with meningococcal septic shock and in organs from porcinis infused with exponentially increasing numbers of *N. meningitidis* (reference strain H44/76) vs. controls.The Z-score indicates predicted activation state of canonical pathways in the tissue samples from meningococcal septic shock patients vs. controls and in organs from porcinis infused with exponentially increasing numbers of *N. meningitidis* (reference strain H44/76) vs. controls. Orange or lighter shades of orange indicate a positive Z-score and upregulation of the pathway. Blue color or lighter shades of blue indicate a negative Z-score and downregulation of the pathway. The transcripts in the gene signaling pathway are expressed as fold change (FC) values. Red or lighter shades of red indicate positive FC-values and upregulation of transcripts; green color or lighter shades of green indicate negative FC-values and downregulation of transcripts. The color gray indicates that a predicted activation state of a gene/transcript in the canonical pathway signaling network is not affected, the gene/transcript was in the dataset but did not pass the analysis cutoffs. White color indicates that gene/transcript are not present in the dataset.

In the top four downregulated canonical pathways (**Figure 3** and **Additional File 4_Table 2**), the top genes involved in downregulated canonical pathways were IL-6, IL1B, CCL2, and NFKBIA. The main differences between the two studies in predicted activation of genes were the observation of expression of IL1RL1, HSP90B1, and TNFAIP3 in the human model and unique genes such as IL1A, IL1RN, TNF, IL-18, and TLR4 in the porcine model.

**Figure 4:**
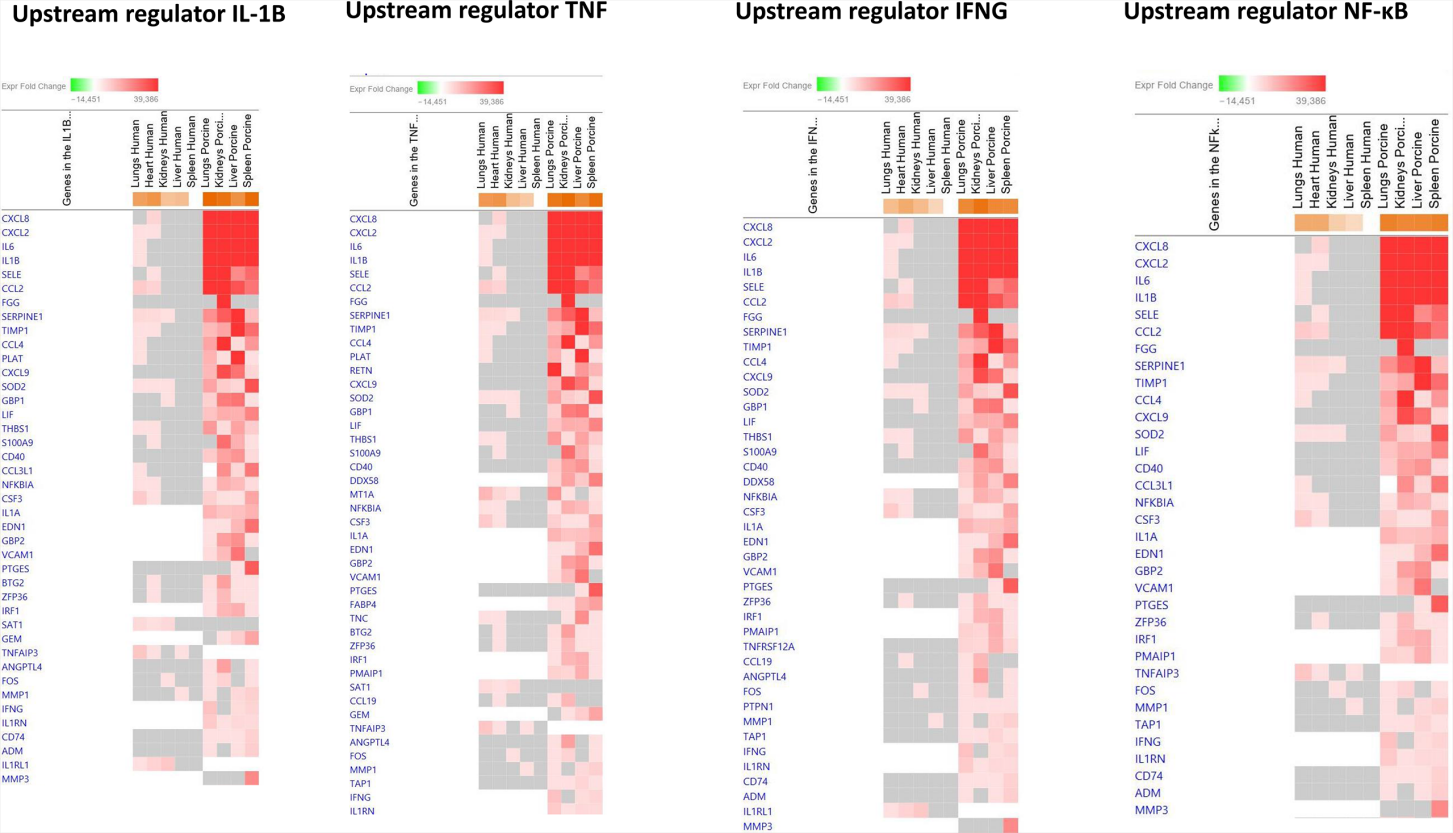
Predicted gene signaling pathways from the top upregulated upstream regulators in FFPE tissue samples from patients with meningococcal septic shock and in organs from porcinis infused with exponentially increasing numbers of *N. meningitidis* (reference strain H44/76) vs. controls. The Z-score indicates predicted activation state of genes in the upstream regulator signaling network. Orange or lighter shades of orange indicate a positive Z-score and upregulation of the upstream regulator. The transcripts in the signaling network are expressed as Fold Change (FC) values. Red or lighter shades of red indicates positive FC-values and upregulation of transcripts; green color or lighter shades of green indicate negative FC-values and downregulation of transcripts. The color gray indicates that a predicted activation state of a gene/transcript in the upstream regulator pathway signaling network is not affected (the gene/transcript was in the dataset but did not pass the analysis cutoffs). White color indicates that gene/transcript are not present in the dataset.

To identify the cascade of upstream transcriptional regulators that can explain the gene expression changes in our datasets, an upstream regulator analysis showed that IL1B, TNF, IFNG, NFκB (complex), IL1A, IL1, OSM (Oncostatin M), TRL3, TRL4, and IL-6 were activated (**Figure 1C** and **Table 6**). The genes involved in these upregulated upstream regulators are mostly the same in the two models: CXCL8 (IL-8), CXCL2 (MIP2-α), IL-6, IL-18, SELE (E-selectin), CCL2 (MCP1), FGG (fibrinogen gamma gene), SERPINE1 (PAI-1), and TIMP (Tissue Inhibitor of Metalloproteinase 1) (**Figure 4** and **Additional File 5 Table 3**).

**Table 6:** Predicted upstream regulators based on Z-score values in FFPE tissue samples from patients with meningococcal septic shock and in organs from porcinis infused with exponentially increasing numbers of *N. meningitidis* (reference strain H44/76) vs. controls.

	Z-score	Z-score	Z-score	Z-score	Z-score	Z-score	Z-score	Z-score	Z-score
Upstream Regulators	Lungs Human	Heart Human	Kidneys Human	Liver Human	Spleen Human	Lungs Porcine	Kidneys Porcine	Liver Porcine	Spleen Porcine
IL1B	5.2	5.8	3.3	3.6	N/A	8.1	7.6	6.0	8.0
TNF	5.5	5.8	3.6	2.9	N/A	7.3	8.0	6.4	7.7
IFNG	3.6	4.6	3.7	2.2	N/A	6.4	7.5	6.4	6.2
NFkB (complex)	4.6	4.6	2.6	2.0	N/A	6.8	6.4	6.5	6.8
IL1A	4.6	4.5	3.5	2.8	N/A	5.3	5.7	5.0	5.8
IL1	4.3	4.1	2.9	2.0	N/A	5.0	5.6	5.2	5.4
OSM	3.6	4.3	3.4	2.8	N/A	5.1	5.7	4.0	5.1
TLR3	3.9	4.0	2.8	2.6	N/A	5.1	4.7	5.1	5.7
TLR4	3.5	3.5	1.7	2.2	N/A	5.8	5.8	6.0	5.3
IL6	3.8	4.5	3.9	2.4	N/A	4.6	5.4	3.4	5.7
RELA	4.0	4.0	1.3	2.4	N/A	5.9	5.3	4.8	5.1
P38 MAPK	3.2	3.9	2.4	2.0	N/A	5.9	5.3	4.5	5.5
F2	3.9	4.2	2.4	2.0	N/A	5.2	5.3	4.4	5.0
CSF2	3.3	4.0	2.4	2.4	N/A	5.8	5.0	4.6	4.5
TLR9	2.9	3.4	2.2	1.7	N/A	5.4	5.2	5.3	5.6
Interferon alpha	2.4	2.9	1.3	2.0	N/A	5.5	5.9	6.1	5.8
AGT	2.6	3.5	2.7	0.6	N/A	5.3	6.0	5.0	4.9
MYD88	3.4	3.9	2.2	N/A	N/A	5.5	5.1	5.3	5.0
EGF	3.6	4.1	2.0	1.9	N/A	4.9	5.1	3.7	5.0
IL2	3.4	2.6	1.4	N/A	N/A	5.7	5.6	5.5	5.6
PDGF BB	2.7	4.1	2.7	2.4	N/A	4.4	4.7	3.1	5.0
APP	3.2	2.6	1.2	1.2	N/A	5.6	5.4	4.3	5.5
TLR7	3.6	3.8	N/A	2.0	N/A	4.7	4.8	5.1	4.7
IKBKB	3.2	3.6	1.8	2.4	N/A	5.0	4.5	4.1	3.8
CD3	3.7	3.7	2.4	0.8	N/A	4.5	4.5	4.3	4.2
CD40LG	2.6	2.9	2.2	2.2	N/A	4.5	4.6	4.2	4.6
STAT1	2.4	3.1	3.0	N/A	N/A	4.1	5.2	5.3	4.4
Jnk	3.1	3.2	2.0	N/A	N/A	5.1	4.7	4.4	4.9
IL33	3.5	3.5	1.3	2.2	N/A	4.7	3.6	3.7	4.5
TICAM1	2.8	3.1	N/A	N/A	N/A	5.4	4.9	5.2	5.4
LDL	2.9	3.2	2.4	0.8	N/A	4.9	4.6	4.5	3.5
Ige	3.4	3.5	N/A	N/A	N/A	4.8	4.6	5.3	5.0
IL17A	3.6	3.9	N/A	0.9	N/A	4.7	5.0	4.3	3.9
IFNA2	3.0	3.6	1.9	N/A	N/A	3.9	4.5	4.7	4.8
NFKB1	2.9	2.6	1.2	2.0	N/A	4.9	4.4	3.8	4.4

≪Comparison analysis≫ of upstream regulators significantly enriched in FFPE tissue samples from lungs, heart, kidneys, liver, and spleen from meningococcal septic shock patients vs. controls patients (acute non-infectious death) and in FF tissue samples from lungs, kidneys, liver, and spleen from porcinis infused with exponentially increasing numbers of *N. meningitidis* (reference strain H44/76) vs. controls were performed. The table shows the top most upregulated upstream regulators ranked according to expression levels in FFPE tissues from lungs. The Z-score indicates predicted activation state of the upstream regulators. Note that only the top upstream regulators are shown. N/A = not applicable.

**Figure 5:**
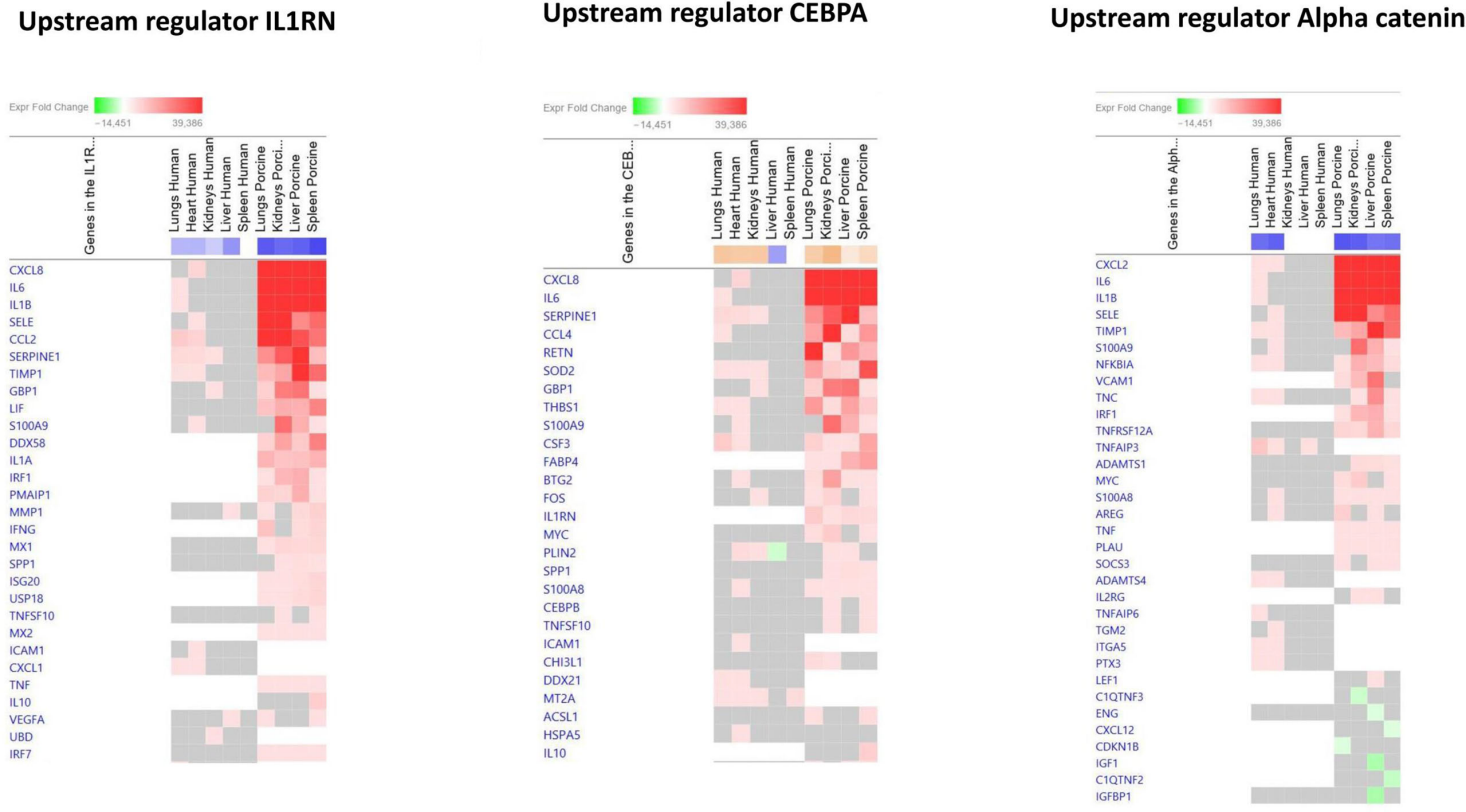
Predicted gene signaling pathways from the top downregulated upstream regulators in FFPE tissue samples from patients with meningococcal septic shock and in organs from porcinis infused with exponentially increasing numbers of *N. meningitidis* (reference strain H44/76) vs. controls. The Z-score indicates predicted activation state of genes in the upstream regulator signaling network. Orange or lighter shades of orange indicate a positive Z-score and upregulation of the upstream regulator signaling network. Blue color or lighter shades of blue indicate a negative Z-score and downregulation of the upstream regulator. The transcripts in the signaling network are expressed as fold change (FC) values. Red or lighter shades of red indicate positive FC-values and upregulation of transcripts; green color or lighter shades of green indicate negative FC-values and downregulation of transcripts. The color gray indicates that a predicted activation state of a gene/transcript in the upstream regulator pathway signaling network is not affected (the gene/transcript was in the dataset but did not pass the analysis cutoffs). White color indicates that gene/transcript are not present in the dataset.

The main downregulated upstream regulators were IL1RN and Alpha catenin (**Figure 5** and **Additional File 6_Table 4**). The genes involved in these downregulated upstream regulators are also largely the same in the two models: CXCL8 (IL-8), CXCL2 (MIP2-alpha), IL-6, SELE (E-selectin), IL1B, SERPINE1 (PAI-1), TIMP1 (Tissue Inhibitor of Metalloproteinase 1), and S100A9 (**Figure 5** and **Additional File 6_Table 4**).

### Cytokine quantification

**Figure 6:**
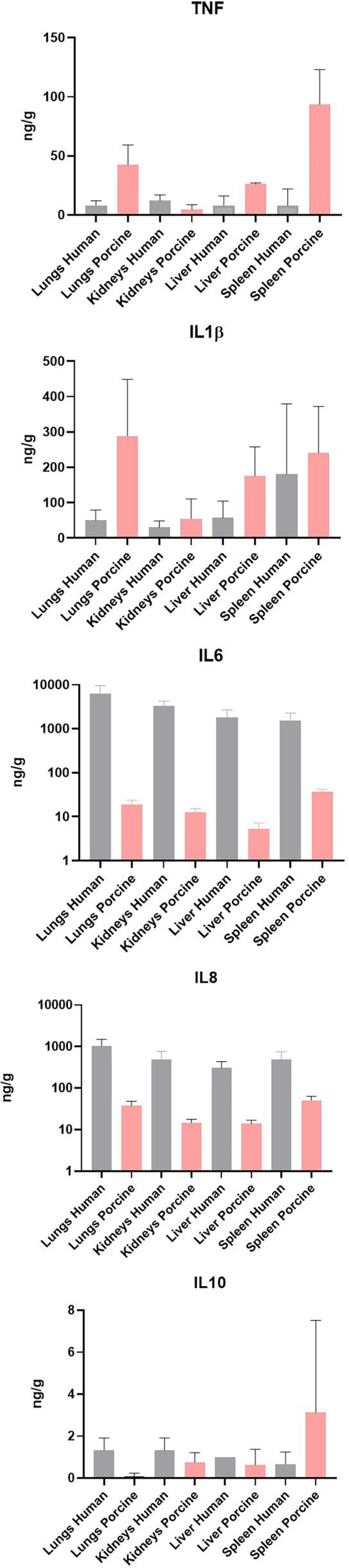
Cytokine concentration in fresh frozen tissue samples from patients with meningococcal septic shock (*n* = 3) and in fresh frozen tissue samples from the experimental porcine model (*n* = 8). The concentration unit is ng/g for the organ samples.

Quantification of selected proteins in FF tissue samples from meningococcal septic shock patients (*n* = 3) and porcine experimental model (*n* = 8) have previously been quantitated in supernatants after homogenization of the organ (Hellerud et al., 2015; Brusletto et al., 2020). Results of the quantification for proteins analyzed in both models are shown in **Figure 6**. All proteins were detected in all organs; however, TNF and IL-1β showed higher levels in the porcine organs than in the patients’ organs, while IL-6 and IL-8 showed the highest levels in humans. The measured IL-10 concentration was almost the same in patients´ and porcine organs.

## Discussion

The results of our porcine model, simulating the exponential growth of *N. meningitidis* in meningococcal septic shock patients, suggest that the transcriptomic changes observed in the patients’ large organs are reflected in the porcine organs. The reason for choosing a porcine model to study meningococcal septic shock is the similarity between the physiological reaction pattern of the circulatory systems of man and porcine (Opal, 1999). Both humans and porcinis are highly sensitive to LPS (Opal, 1999). This has been confirmed in four different series of porcine experiments (Nielsen et al., 2009; Hellerud et al., 2010; Hellerud et al., 2015; Hellerud et al., 2017). We have studied the effect of *N. meningitidis* on the cardiovascular and pulmonary systems together with the effects of the bacteria on kidneys, liver, and spleen. In the porcinis, we monitored the immune responses, the activation and subsequently the inhibition of the coagulation systems and the upregulation of the complement system in the blood and large organs. We compared the altered gene expressions at the mRNA level of the key cytokines, chemokines, and other proteins related to inflammation in plasma and tissues of the organs. They all play a major role in the pathophysiology of meningococcal septic shock and multiorgan failure. We have previously documented that LPS is the most potent but not the only group of meningococcal molecules, which induce inflammation in the porcinis, which was reflected in the gene activation (Hellerud et al., 2010).

We used the porcine model with doubling doses of the bacteria infused intravenously every 30 min, simulating the growth of meningococci in the circulation and large organs as observed in patients (Brandtzaeg et al., 1989a; van Deuren et al., 2000; Hackett et al., 2002; Ovstebo et al., 2004; Darton et al., 2009; Brandtzaeg and van Deuren, 2012). During the 4-h-long experiment, we were able to monitor the animals continuously and collect enough blood samples for longitudinal monitoring of numerous mediators. Immediately after euthanizing the animals, we could collect autopsy tissue specimens for further studies.

It is essential that the animal model of meningococcal septic shock reflects the distribution of meningococci and inflammatory responses in different organs at a transcriptomic level. The present study is an extension of our previous study of transcriptomic changes of the large organs found in five patients with lethal meningococcal septic shock and multiple organ failure (Brusletto et al., 2020). To our knowledge, no other study of acute human septic shock leading to multiple organ failure and severe coagulopathy has compared the changes in the transcriptomic gene expressions in the large organs with those obtained in a large animal model infused with the same bacterium.

When comparing the predicted Z-scores of biofunctions, canonical pathways, and upstream regulators of the organs in the porcine model with the genes upregulated in humans, we see that the porcine model closely reflects the changes found in patients (**Figure 1A** and **Table 4**). Of 32 biofunctions, 30 were upregulated in the patients and the porcinis. Organismal death, morbidity, and mortality were downregulated in the patients and porcinis, indicating close similarity in regulation of the biofunctions (**Figure 1A** and **Table 4**).

Of the patients’ lung samples, of 18 canonical pathways meeting the selection criteria, 15 were upregulated and 2 (LXR/RXR Activation and PPARα/RXRα Activation) were downregulated in both patients and porcinis (**Figure 1B** and **Table 5**). Only one pathway (NF-κB signaling) was downregulated in the patients and upregulated in the porcinis (**Figure 1B** and **Table 5**). Thirty-five upstream regulators in lung samples from both models revealed a similar upregulation pattern (**Figure 1C** and **Table 6**). Thus, of 85 biofunctions, canonical pathways, and upstream regulators examined in patients, only 1 was downregulated in patients and upregulated in the porcinis. Although the changes are more pronounced in porcine tissues, the tendency is the same (**Figure 1**).

We have previously demonstrated that, although massive intravascular growth of meningococci in the circulation is regarded to be a key element of fulminant meningococcal sepsis, solid organs are also highly involved in the inflammatory responses in patients with this disease (Hellerud et al., 2015; Brusletto et al., 2017; Brusletto et al., 2020).

Our results suggest that a porcine model simulating fulminant meningococcal sepsis can be a valuable tool in studying the pathophysiological of this often fatal disease in humans. Also, possible interventional aspects that cannot be easily studied in humans due to the disease rarity and abrupt development may be elucidated. A highly upregulated systemic inflammation driven by a wide variety of inflammatory mediators involving thousands of genes is the key element of the meningococcal septic shock.

Quantification of meningococci in the organs reveals accumulation of meningococci in all tissues examined, with the highest levels being detected in the lungs in both the patients and the porcine model (Hellerud et al., 2015; Brusletto et al., 2017; Brusletto et al., 2020). However, the measured numbers of meningococci in the porcine lungs and liver were higher than observed in the similar organs of the patients. The difference presumably relates to the experimental conditions in the porcine model. The infusion site, the concentration of bacteria, and the logarithmically increasing velocity of the infusion may lead to entrapment of large numbers of bacteria in the liver and the lungs after intravenous administration for 4 h (Nielsen et al., 2009). Patients with meningococcal septic shock have a median time from onset of the symptoms to hospital admission of 12 h (van Deuren et al., 2000; Brandtzaeg and van Deuren, 2012). This more gradual growth of meningococci in the patients’ circulation may possibly lead to a more equal seeding of the patients’ organs than observed in the porcine model. Possible differences in the distribution of macrophages between human and porcinis may also play a role in the concentrations of bacteria in the organs.

It is an important observation that the highest numbers of bacteria are found in the lungs in both the patients and the porcinis. This implies that the lungs have a significant functional capacity to attract large numbers of circulating bacteria in patients with meningococcal septic shock as well as in porcinis used in these experiments, although *N. meningitidis* is not a porcine pathogen. Moreover, the organ distribution of *N. meningitidis* in the patients is reflected well in the porcine organs. However, there are some marked individual differences between patients and porcinis in this experiment. The key inflammatory cytokines IL-6 and IL-8 are much higher in the patients’ organs than in the porcinis’ organs (**Figure 6**).

In tissues collected from the patients’ hearts, the numbers of meningococci were almost as high as detected in the lungs (Brusletto et al., 2020). In total, 2029 genes were differentially expressed in the human heart as compared with 2039 differentially expressed genes in the lungs (Brusletto et al., 2020). We assume that a similar picture may be present in the porcinis’ hearts, but unfortunately, we lack data to support this assumption. Tissue samples from the porcinis’ hearts were not included in the porcine experiments at the time, as the primary focus of the porcine model was examination of organs containing a large number of macrophages.

Questions can be raised concerning the comparison of gene expression in different tissues stored at different temperatures for several years. FFPE tissues from patients were stored at room temperature (20°C), whereas additional FF tissues from three of these patients and tissues from the pigs were frozen (−80°C). We have earlier investigated the influence of storage temperatures on the transcriptional profiles in samples stored from 2 to 6 years. The results showed a good consistency between the methods of storage (Brusletto et al., 2020).

The measured gene transcription levels were generally higher in the porcine organs than in the organs from the patients. The higher levels can be related to higher levels of bacteria as discussed earlier, but also to interspecies differences regarding immune responses and sensitivity to bacterial antigens, primarily LPS. Also, the compressed time course (4 h) of the experiments may have an impact. The patients had recognized symptoms less than 24 h before hospital admission and died within 4.5 h after admission. It has previously been shown that the plasma levels of key cytokines in meningococcal sepsis, including TNF, IL-1β, IL-6, IL-8, and IL-10, are rapidly downregulated after the first dose of effective antibiotics is given (Brandtzaeg et al., 1989a; van Deuren, 1994; Brandtzaeg, 1995; Brandtzaeg et al., 2001). The cytokines decline in parallel with declining plasma LPS levels. The median half-life of LPS in meningococcal septic shock plasma is 2 h after receiving antibiotics (Brandtzaeg et al., 1989a; Brandtzaeg et al., 1995; Brandtzaeg et al., 1996; Frieling et al., 1996; Brandtzaeg et al., 2001). The decreasing levels of plasma cytokines primarily reflect downregulation of intracellular cytokine production presumably induced by downregulation of specific gene transcription that varies from organ to organ (**Figure 1**). Since the patients in this study lived from 1 to 4.5 h after receiving antibiotics, the intracellular mRNA for the cytokines were presumably downregulated when they died. Autopsies of the patients were performed within 24 h. The porcinis were euthanized 4 h after the start of the experiment and autopsied, and the tissues were collected immediately after and frozen at −80°C. The abrupt immune response triggered in the porcinis may partly explain the higher levels of mRNA found in the porcinis than in the patients. Species differences may also contribute to discrepancy in levels of gene expressions. However, the general picture indicates that the up- or downregulation occurred in the same direction for hundreds of genes, suggesting great similarity in gene regulation (**Figures 1**–**3**).

Some of the differences in the transcripts may also be related to differences in the micro arrays, since the human array includes both coding and non-coding RNA, while in the porcine array, only coding RNA is included. However, IPA provides a comprehensive database of known transcripts, networks, and pathways that are continuously being updated based on published work on gene functions and interactions.

Despite the differences in bacterial numbers in the porcine and human organs, and the overall higher level of transcriptional activation in the porcinis, there was a striking concordance between the patients and the porcinis regarding the pattern of transcriptional activation and activated pathways. Comparison analysis demonstrated similar patterns of upregulation of genes being associated with a large range of inflammatory biofunctions in the patients and the porcine model (**Figure 1A** and **Table 4**). On the contrary, genes associated with biofunctions such as organismal death, morbidity, and mortality were similarly downregulated in the patients and the porcine model (**Figure 1A** and **Table 4**). Comparison analysis of main predicted canonical pathways also demonstrated a high degree of similarity regarding up- and downregulation (**Figure 1B**, **Table 5**, **Figure 2**, **Figure 3**, **Additional File 3_Table 1**, and **Additional File 4_Table 2**). Of interest, NF-kB signaling differed from this general pattern, being downregulated in the patients´ lungs but upregulated in the porcine organs. A possible reason for this can be that the patients were given antibiotics prior to their death as discussed above.

One of the main predicted canonical pathways in both models was IL-17 Signaling, a pathway that appears to be highly involved in the pathogenesis of sepsis. It has been shown that excessive IL-17A production disrupts immune homeostasis and contributes to the development and progression of sepsis (Ge et al., 2020). Finally, comparison analyses for specific upstream regulators confirmed the high degree of similarity of the inflammatory response, applying to up- as well as downregulated individual genes **Figure 1C**, **4**, **5**, **Table 6**, **Additional File 5_Table 3**, and **Additional File 6_Table 4**).

A key feature of fulminant meningococcal disease is that despite the general pattern with inflammatory responses associated with survival of the organism being upregulated and responses associated with organismal death being downregulated, the outcome of this disease is often fatal. The large number of pro-inflammatory mechanisms that are activated during meningococcal infection appear to be beneficial to the host when activated at a comparatively low level (LPS <7 endotoxin units (EU)/ml plasma) (Brandtzaeg et al., 1989a; Ovstebo et al., 2004; Hellerud et al., 2008; Ovstebø et al., 2008). However, when the inflammatory responses pass certain levels, induced by the increasing load of meningococci, it becomes detrimental to the patient because of the lack of limitation and involvement of the whole body. The driving force is the bacterial proliferation surpassing certain critical levels (10^6^/*N. meningitidis* DNA copies/ml plasma) (Hackett et al., 2002; Ovstebo et al., 2004; Hellerud et al., 2008; Darton et al., 2009; Brandtzaeg and van Deuren, 2012).

Core analysis of each organ from the patients revealed different top upstream regulators for all the organs (**Table 3**). Pro-inflammatory regulators were mostly activated in the lungs, while in the other organs, mostly transcription factors that regulate signaling pathways involved in development, growth, repair, and homeostasis, and triglyceride synthesis were activated. These results contrasted with the results of the porcine model, where the activated top upstream regulators were pro-inflammatory in all organs, and the main top upstream regulator in all organs proved to be IL-1B followed by other key inflammatory regulators like TNF, IL-6, and TGFB1. This difference may possibly be explained by a more intense inflammatory stimulation of the porcinis’ organs within a shorter time frame than was the case with the patients.

Core canonical pathway analysis revealed few canonical pathways in the liver and spleen in the meningococcal septic shock patient group (**Additional File 1_Figure S1**). These results contrasted the changes observed in the porcine model, where several canonical pathways were identified in these organs (**Additional File 2_Figure S2**). The only organs with a range of canonical pathways identified in the patients were the lungs, heart, and kidneys (**Additional File 1_Figure S1**). Taken together, these results may indicate that the role of individual organs in the inflammatory response in meningococcal sepsis differs somewhat in patients as compared with the porcine model. However, the results regarding the human spleen and liver may have been confounded by higher level of RNA degradation in these organs than in the lungs, heart, and kidneys (Bauer, 2007; Zhu et al., 2017; Ferreira et al., 2018; Scrivano et al., 2019). Also, immune hibernation in the spleen as observed in the course of sepsis (Levy, 2007; Azevedo, 2010) may play a role in the seemingly low activation seen in the spleen from the patients (Brusletto et al., 2020).

Quantification of selected key proteins was detected in all organs in both models. The levels of the cytokines IL-6 and IL-8 in the meningococcal septic shock patients’ organs were, however, higher compared with the porcine experimental model (**Figure 6**). The reason for this may be a more profound post-translation regulation of cytokine production or protein degradation regulation. It has been shown that genome-wide correlation between expression levels of mRNA and protein are quite poor, approximately 40% explanatory power across many studies. The discrepancy is usually ascribed to other levels of regulation between transcript and protein product (de Sousa Abreu et al., 2009; Vogel and Marcotte, 2012; Koussounadis et al., 2015).

A weakness of our model is that the experiment simulating the exponential growth of meningococci, with an estimated duplication time of 30 min, is compressed to 4 h. In real life, the proliferation of meningococci in the shock patients is ongoing longer than 4 h, given the very high levels of *N. meningitis* DNA as detected by PCR (10^6^–10^8^ copy number/ml) in plasma on hospital admission (Hackett et al., 2002; Ovstebo et al., 2004; Darton et al., 2009). The median time between disease onset and hospital admission for meningococcal sepsis with persistent shock was 12–13 h in different studies supporting this conclusion (Brandtzaeg et al., 1989a; van Deuren et al., 2000; de Greeff et al., 2008). The time span of 4 h for our experiment was chosen for logistical and technical reasons. A future porcine model of meningococcal septic shock lasting 12 h and starting with a much lower dose of meningococci would induce more lenient physiological and immunological responses and simulate the human disease more correctly. A model developed according to these principles could be a suitable tool to evaluate in more details the physiological, immunological, and transcriptomic changes of fulminant meningococcal septicemia with persistent shock, heart, pulmonary, and renal failure combined with disseminated intravascular coagulation. Death or severe sequelae of this dreaded infection is primarily related to these complications.

## Conclusion

This study demonstrates that the immune activation measured at the transcriptomic level in specific organs in patients with fulminant meningococcal sepsis is closely reproduced in a porcine model of the disease. This implies that such a revised porcine model can reproduce important immunological mechanisms of this infection and be a valuable tool in further investigating inflammatory aspects of the disease and possible treatment options.

## Data availability statement

The original contributions presented in the study are included in the article/**Supplementary Material**. Further inquiries can be directed to the corresponding author. The datasets supporting the conclusions of this article are available in the Gene Expression Omnibus (GEO) repository https://www.ncbi.nlm.nih.gov/geo/ under the identifier GSE141864 and GSE198668 in accordance with minimum information about a microarray experiment (MIAME) standard.

## Ethics statement

The studies involving human participants were reviewed and approved by the Regional Medical Ethical Committee of South East Norway (2011/1413C “Translational research, meningococcal disease” and 2011/753 “ Studies of invasive meningococcal and pneumococcal disease”). The patients’ samples were collected after informed consent from patient parents or relatives and according to the Helsinki declaration. The Director of Public Prosecutions approved the use of forensic material for this research. Written informed consent to participate in this study was provided by the participants’ legal guardian/next of kin. The animal study was reviewed and approved by the Norwegian Animal Research Authority, and animals were treated according to the Norwegian Laboratory Animal Regulations. The porcine experiments were performed in accordance to the Norwegian laboratory animal regulations and the University Animal Care Committee approved the protocol.

## Author contributions

Study concept and design: BB, RØ, PB, and BH; performed laboratory experiments: BB and BH; performed statistical analysis and drafted the manuscript: BB, RØ, BH, OO, and PB; performed data analysis: BB and OO; critical revision of the manuscript: BB, RØ, PB, BH, and OO. All authors read and approved the final manuscript.

## Funding

This research was funded by South –Eastern Norway Regional Health Authority program and Norwegian Research Council.

## Conflict of interest

The authors declare that the research was conducted in the absence of any commercial or financial relationships that could be construed as a potential conflict of interest.

## Publisher’s note

All claims expressed in this article are solely those of the authors and do not necessarily represent those of their affiliated organizations, or those of the publisher, the editors and the reviewers. Any product that may be evaluated in this article, or claim that may be made by its manufacturer, is not guaranteed or endorsed by the publisher.

## Supplementary material

Supplementary Figure 1_1 Transcriptional profiles of canonical pathways in FFPE tissue samples from meningococcal septic shock patients. The figure displays the top canonical pathways enriched in each organ. A ≪core analysis≫ with FC ≥ |2.0| and *p*-value < 0.05 was performed separately for each organ. Significantly enriched canonical pathways were identified with a right-tailed Fisher’s exact test *p* < 0.05, after correction for multiple testing using the Benjamini-Hochberg method. The Z-score = | ± 2| indicates predicted activation state of canonical pathway. Blue color or lighter shades of blue indicate a negative Z-score and down-regulation of the pathway, and orange or lighter shades of orange indicate a positive Z-score and up-regulation of the pathway. Gray color indicates no activity pattern available. Z-score value >| ± 2| are displayed. Z-score limit 2 for lungs, heart, and kidneys. For liver, Z-score 1, and for spleen, no Z-score limit. Pathways that are not involved for that particular organ or disease are filtered away.

Supplementary Figure 2_2 Transcriptional profiles of canonical pathways in fresh frozen (FF) tissue samples from porcinis infused exponentially increasing numbers of *N. meningitidis* (reference strain H44/76). The figure displays the top canonical pathways enriched in each organ. A ≪core analysis≫ with FC ≥ |2.0| and *p*-value < 0.05 was performed separately for each organ. Significantly enriched canonical pathways were identified with a right-tailed Fisher’s exact test *p* < 0.05, after correction for multiple testing using the Benjamini–Hochberg method. The Z-score=| ± 2| indicates predicted activation state of canonical pathway. Blue color or lighter shades of blue indicate a negative Z-score and down-regulation of the pathway, and orange or lighter shades of orange indicate a positive Z-score and up-regulation of the pathway. Gray color indicates no activity pattern available. Z-score value >| ± 2| are displayed. For all organs showing canonical pathways corrected by *p* < 0.05, FC2. BH 1.3 corrected false *p*-value. Z-score limit 2. Pathways that are not involved for that particular organ or disease are filtered away. This figure shows the top 12 most activated pathways.

## Abbreviations

CCL2, C-C motif chemokine ligand 2 (alias MCP1); CEBPA, CCAAT enhancer binding protein alpha; *ctrA*, capsule transport A; CXCL1, chemokine (C-X-C motif) ligand 1 (alias GRO1); CXCL2, chemokine (C-X-C motif) ligand 2 (alias MIP-2α); CXCL8, chemokine (C-X-C motif) ligand 8 (alias IL-8); DIC, disseminated intravascular coagulation; EDN1, endothelin 1; EIF2, eukaryotic Initiation factor 2; FC, fold changes; FDR, false discovery rate; FF, fresh frozen; FFPE, formalin-fixed, paraffin-embedded; FGG, fibrinogen gamma gene; G-CSF, granulocyte-colony stimulating factor (alias CFR 3); GEO, Gene Expression Omnibus; HMBG1, High mobility group box 1 protein; 1; HSP90B1, Heat shock protein 90kDa beta member 1; ICAM, intercellular adhesion molecules (alias CD 54); IFNG, Interferon gamma; IL, interleukin; IL1RN, interleukin-1 receptor antagonist (IL-1RA); IL1RL1, Interleukin 1 receptor-like 1 (ST2); IPA, ingenuity pathway analysis; ITGA5, integrin alpha-5; LPS, lipopolysaccharides; LXR, liver X receptor; MCP-1, monocyte chemoattractant protein-1 (alias CCL2); MIAME, minimum information about a microarray experiment; MSS, meningococcal septic shock; NF-κB, nuclear factor kappa-light-chain-enhancer of activated B cells; NFKB1A, NF-kappa-B inhibitor alpha; NmDNA, *N. meningitidis* DNA; OSM, oncostatin M; PAI-1, plasminogen activator inhibitor-1 (alias SEPINE 1); PPAR, peroxisome proliferator-activated receptors; RXR, retinoid X receptor; S100A9, calcium binding protein A9 (calgranulin B); RT, room temperature; SELE, E-selectin; TF, tissue factor; TIMP, tissue inhibitor of metalloproteinase 1; TLRs, toll-like receptor family; TNF, tumor necrosis factor; TNFAIP3, tumor necrosis factor, alpha-induced protein 3; TREM1, triggering receptor expressed on myeloid cells 1; VCAM-1, vascular cell adhesion molecule 1; VIP, vasoactive intestinal peptide.

## Glossary

**Table:**

CCL2	C-C motif chemokine ligand 2 (alias MCP1)
CEBPA	CCAAT enhancer binding protein alpha
*ctrA*	capsule transport A
CXCL1	chemokine (C-X-C motif) ligand 1 (alias GRO1)
CXCL2	chemokine (C-X-C motif) ligand 2 (alias MIP-2α)
CXCL8	chemokine (C-X-C motif) ligand 8 (alias IL-8)
DIC	disseminated intravascular coagulation
EDN1	endothelin 1
EIF2	eukaryotic Initiation factor 2
FC	fold changes
FDR	false discovery rate
FF	fresh frozen
FFPE	formalin-fixed, paraffin-embedded
FGG	fibrinogen gamma gene
G-CSF	granulocyte-colony stimulating factor (alias CFR 3)
GEO	Gene Expression Omnibus
HMBG1	High mobility group box 1 protein, 1
HSP90B1	Heat shock protein 90kDa beta member 1
ICAM	intercellular adhesion molecules (alias CD 54)
IFNG	Interferon gamma
IL	interleukin
IL1RN	interleukin-1 receptor antagonist (IL-1RA)
IL1RL1	Interleukin 1 receptor-like 1 (ST2)
IPA	ingenuity pathway analysis
ITGA5	integrin alpha-5
LPS	lipopolysaccharides
LXR	liver X receptor
MCP-1	monocyte chemoattractant protein-1 (alias CCL2)
MIAME	minimum information about a microarray experiment
MSS	meningococcal septic shock
NF-κB	nuclear factor kappa-light-chain-enhancer of activated B cells
NFKB1A	NF-kappa-B inhibitor alpha
NmDNA	*N. meningitidis* DNA
OSM	oncostatin M
PAI-1	plasminogen activator inhibitor-1 (alias SEPINE 1)
PPAR	peroxisome proliferator-activated receptors
RXR	retinoid X receptor
S100A9	calcium binding protein A9 (calgranulin B)
RT	room temperature
SELE	E-selectin
TF	tissue factor
TIMP	tissue inhibitor of metalloproteinase 1
TLRs	toll-like receptor family
TNF	tumor necrosis factor
TNFAIP3	tumor necrosis factor, alpha-induced protein 3
TREM1	triggering receptor expressed on myeloid cells 1
VCAM-1	vascular cell adhesion molecule 1
VIP	vasoactive intestinal peptide.

## References

[B1] Azevedo L. C. (2010). Mitochondrial dysfunction during sepsis. Endocr. Metab. Immune Disord. Drug Targets 10 (3), 214–223. doi: 10.2174/187153010791936946 20509844

[B2] Bauer M. (2007). RNA In forensic science. Forensic. Sci. Int. Genet. 1 (1), 69–74. doi: 10.1016/j.fsigen.2006.11.002 19083730

[B3] Bjune G. Høiby E. A. Grønnesby J. K. Arnesen O. Fredriksen J. H. Halstensen A.. (1991). Effect of outer membrane vesicle vaccine against group b meningococcal disease in Norway. Lancet 338 (8775), 1093–1096. doi: 10.1016/0140-6736(91)91961-s 1682541

[B4] Brandtzaeg P. (1995). “"Pathogenesis of meningococcal infections,",” in Meningococcal disease. Ed. Cartwright K. (Chichester: John Wiley & Sons), 71–114.

[B5] Brandtzaeg P. (2006). “"Pathogenesis and pathophysiology of invasive meningococcal disease,",” in Handbook of meningococcal disease: Infection biology, vaccination, clinical management. Eds. Frosch M. Maiden M. C. J. (Weinheim: Wiley-VCH Verlag GmbH & Co), 427–480.

[B6] Brandtzaeg P. Bjerre A. Ovstebo R. Brusletto B. Joo G. B. Kierulf P. (2001). Neisseria meningitidis lipopolysaccharides in human pathology. J. Endotoxin. Res. 7 (6), 401–420. doi: 10.1179/096805101101533016 11753210

[B7] Brandtzaeg P. Hogasen K. Kierulf P. Mollnes T. E. (1996). The excessive complement activation in fulminant meningococcal septicemia is predominantly caused by alternative pathway activation. J. Infect. Dis. 173 (3), 647–655. doi: 10.1093/infdis/173.3.647 8627028

[B8] Brandtzaeg P. Joo G. B. Brusletto B. Kierulf P. (1990). Plasminogen activator inhibitor 1 and 2, alpha-2-antiplasmin, plasminogen, and endotoxin levels in systemic meningococcal disease. Thromb. Res. 57 (2), 271–278. doi: 10.1016/0049-3848(90)90326-8 2315889

[B9] Brandtzaeg P. Kierulf P. Gaustad P. Skulberg A. Bruun J. N. Halvorsen S.. (1989a). Plasma endotoxin as a predictor of multiple organ failure and death in systemic meningococcal disease. J. Infect. Dis. 159 (2), 195–204. doi: 10.1093/infdis/159.2.195 2492587

[B10] Brandtzaeg P. Mollnes T. E. Kierulf P. (1989b). Complement activation and endotoxin levels in systemic meningococcal disease. J. Infect. Dis. 160 (1), 58–65. doi: 10.1093/infdis/160.1.58 2471750

[B11] Brandtzaeg P. Oktedalen O. Kierulf P. Opstad P. K. (1989c). Elevated VIP and endotoxin plasma levels in human gram-negative septic shock. Regul. Pept. 24 (1), 37–44. doi: 10.1016/0167-0115(89)90209-7 2500680

[B12] Brandtzaeg P. Ovstebø R. Kierulf P. (1995). Bacteremia and compartmentalization of LPS in meningococcal disease. Prog. Clin. Biol. Res. 392, 219–233. 8524927

[B13] Brandtzaeg P. Sandset P. M. Joo G. B. Ovstebo R. Abildgaard U. Kierulf P. (1989d). The quantitative association of plasma endotoxin, antithrombin, protein c, extrinsic pathway inhibitor and fibrinopeptide a in systemic meningococcal disease. Thromb. Res. 55 (4), 459–470. doi: 10.1016/0049-3848(89)90054-6 2510354

[B14] Brandtzaeg P. van Deuren M. (2012). Classification and pathogenesis of meningococcal infections. Methods Mol. Biol. 799, 21–35. doi: 10.1007/978-1-61779-346-2_2 21993637

[B15] Brazma A. Hingamp P. Quackenbush J. Sherlock G. Spellman P. Stoeckert C.. (2001). Minimum information about a microarray experiment (MIAME)-toward standards for microarray data. Nat. Genet. 29 (4), 365–371. doi: 10.1038/ng1201-365 11726920

[B16] Brusletto B. S. Hellerud B. C. Loberg E. M. Goverud I. L. Vege A. Berg J. P.. (2017). Traceability and distribution of neisseria meningitidis DNA in archived post mortem tissue samples from patients with systemic meningococcal disease. BMC Clin. Pathol. 17, 10. doi: 10.1186/s12907-017-0049-9 28824331 PMC5559868

[B17] Brusletto B. S. Loberg E. M. Hellerud B. C. Goverud I. L. Berg J. P. Olstad O. K.. (2020). Extensive changes in transcriptomic "Fingerprints" and immunological cells in the Large organs of patients dying of acute septic shock and multiple organ failure caused by neisseria meningitidis. Front. Cell Infect. Microbiol. 10. doi: 10.3389/fcimb.2020.00042 PMC7045056 32154187

[B18] D'Agati V. C. Marangoni B. A. (1945). The Waterhouse-friderichsen syndrome. New Engl. J. Med. 232 (1), 1–7. doi: 10.1056/nejm194501042320101

[B19] Darton T. Guiver M. Naylor S. Jack D. L. Kaczmarski E. B. Borrow R.. (2009). Severity of meningococcal disease associated with genomic bacterial load. Clin. Infect. Dis. 48 (5), 587–594. doi: 10.1086/596707 19191644

[B20] de Greeff S. C. de Melker H. E. Schouls L. M. Spanjaard L. van Deuren M. (2008). Pre-admission clinical course of meningococcal disease and opportunities for the earlier start of appropriate intervention: a prospective epidemiological study on 752 patients in the Netherlands 2003-2005. Eur. J. Clin. Microbiol. Infect. Dis. 27 (10), 985–992. doi: 10.1007/s10096-008-0535-1 18493804

[B21] de Kleijn E. D. Hazelzet J. A. Kornelisse R. F. de Groot R. (1998). Pathophysiology of meningococcal sepsis in children. Eur. J. Pediatr. 157 (11), 869–880. doi: 10.1007/s004310050958 9835428

[B22] de Sousa Abreu R. Penalva L. O. Marcotte E. M. Vogel C. (2009). Global signatures of protein and mRNA expression levels. Mol. Biosyst. 5 (12), 1512–1526. doi: 10.1039/b908315d 20023718 PMC4089977

[B23] Dretler A. W. Rouphael N. G. Stephens D. S. (2018). Progress toward the global control of neisseria meningitidis: 21st century vaccines, current guidelines, and challenges for future vaccine development. Hum. Vaccin. Immunother. 14 (5), 1146–1160. doi: 10.1080/21645515.2018.1451810 29543582 PMC6067816

[B24] Ferguson J. H. Chapman O. D. (1948). Fulminating meningococcic infections and the so-called Waterhouse-friderichsen syndrome. Am. J. Pathol. 24 (4), 763–795. 18874411 PMC1942741

[B25] Ferreira P. G. Muñoz-Aguirre M. Reverter F. Sá Godinho C. P. Sousa A. Amadoz A.. (2018). The effects of death and post-mortem cold ischemia on human tissue transcriptomes. Nat. Commun. 9 (1), 490. doi: 10.1038/s41467-017-02772-x 29440659 PMC5811508

[B26] Frieling J. T. van Deuren M. Wijdenes J. van Dalen R. Bartelink A. K. van der Linden C. J.. (1996). Interleukin-6 and its soluble receptor during acute meningococcal infections: effect of plasma or whole blood exchange. Crit. Care Med. 24 (11), 1801–1805. doi: 10.1097/00003246-199611000-00007 8917028

[B27] Frosch M. Muller D. Bousset K. Muller A. (1992). Conserved outer membrane protein of neisseria meningitidis involved in capsule expression. Infect. Immun. 60 (3), 798–803. doi: 10.1128/iai.60.3.798-803.1992 1371768 PMC257557

[B28] Ge Y. Huang M. Yao Y. M. (2020). Biology of interleukin-17 and its pathophysiological significance in sepsis. Front. Immunol. 11. doi: 10.3389/fimmu.2020.01558 PMC7399097 32849528

[B29] Gopinathan U. Brusletto B. S. Olstad O. K. Kierulf P. Berg J. P. Brandtzaeg P.. (2015). IL-10 immunodepletion from meningococcal sepsis plasma induces extensive changes in gene expression and cytokine release in stimulated human monocytes. Innate. Immun. 21 (4), 429–449. doi: 10.1177/1753425914547743 25233959

[B30] Gopinathan U. Ovstebo R. Olstad O. K. Brusletto B. Dalsbotten Aass H. C. Kierulf P.. (2012). Global effect of interleukin-10 on the transcriptional profile induced by neisseria meningitidis in human monocytes. Infect. Immun. 80 (11), 4046–4054. doi: 10.1128/iai.00386-12 22966040 PMC3486056

[B31] Hackett S. J. Guiver M. Marsh J. Sills J. A. Thomson A. P. Kaczmarski E. B.. (2002). Meningococcal bacterial DNA load at presentation correlates with disease severity. Arch. Dis. Child 86 (1), 44–46. doi: 10.1136/adc.86.1.44 11806883 PMC1719043

[B32] Hazelzet J. A. Risseeuw-Appel I. M. Kornelisse R. F. Hop W. C. Dekker I. Joosten K. F.. (1996). Age-related differences in outcome and severity of DIC in children with septic shock and purpura. Thromb. Haemost. 76 (6), 932–938. doi: 10.1055/s-0038-1650688 8972013

[B33] Hellerud B. C. Nielsen E. W. Thorgersen E. B. Lindstad J. K. Pharo A. Tonnessen T. I.. (2010). Dissecting the effects of lipopolysaccharides from nonlipopolysaccharide molecules in experimental porcine meningococcal sepsis. Crit. Care Med. 38 (6), 1467–1474. doi: 10.1097/CCM.0b013e3181de8c94 20400898

[B34] Hellerud B. C. Olstad O. K. Nielsen E. W. Troseid A. M. Skadberg O. Thorgersen E. B.. (2015). Massive organ inflammation in experimental and in clinical meningococcal septic shock. Shock 44 (5), 458–469. doi: 10.1097/shk.0000000000000441 26473439

[B35] Hellerud B. C. Orrem H. L. Dybwik K. Pischke S. E. Baratt-Due A. Castellheim A.. (2017). Combined inhibition of C5 and CD14 efficiently attenuated the inflammatory response in a porcine model of meningococcal sepsis. J. Intensive Care 5, 21. doi: 10.1186/s40560-017-0217-0 28261486 PMC5327570

[B36] Hellerud B. C. Stenvik J. Espevik T. Lambris J. D. Mollnes T. E. Brandtzaeg P. (2008). Stages of meningococcal sepsis simulated *in vitro*, with emphasis on complement and toll-like receptor activation. Infect. Immun. 76 (9), 4183–4189. doi: 10.1128/iai.00195-08 18591229 PMC2519403

[B37] Hellum M. Ovstebo R. Brusletto B. S. Berg J. P. Brandtzaeg P. Henriksson C. E. (2014). Microparticle-associated tissue factor activity correlates with plasma levels of bacterial lipopolysaccharides in meningococcal septic shock. Thromb. Res. 133 (3), 507–514. doi: 10.1016/j.thromres.2013.12.031 24423888

[B38] Holten E. (1979). Serotypes of neisseria meningitidis isolated from patients in Norway during the first six months of 1978. J. Clin. Microbiol. 9 (2), 186–188. doi: 10.1128/jcm.9.2.186-188.1979 107188 PMC272987

[B39] Kornelisse R. F. Hazelzet J. A. Savelkoul H. F. Hop W. C. Suur M. H. Borsboom A. N.. (1996). The relationship between plasminogen activator inhibitor-1 and proinflammatory and counterinflammatory mediators in children with meningococcal septic shock. J. Infect. Dis. 173 (5), 1148–1156. doi: 10.1093/infdis/173.5.1148 8627066

[B40] Koussounadis A. Langdon S. P. Um I. H. Harrison D. J. Smith V. A. (2015). Relationship between differentially expressed mRNA and mRNA-protein correlations in a xenograft model system. Sci. Rep. 5 (1), 10775. doi: 10.1038/srep10775 26053859 PMC4459080

[B41] Levy R. J. (2007). Mitochondrial dysfunction, bioenergetic impairment, and metabolic down-regulation in sepsis. Shock 28 (1), 24–28. doi: 10.1097/01.shk.0000235089.30550.2d 17483747

[B42] Martland H. S. (1944). Fulminating meningococcic infection with bilateral massive adrenal hemorrhage (The Waterhouse friderichsen syndrome). J. Nerv. Ment. Dis. 100 (5), 532–533. doi: 10.1097/00005053-194411000-00043

[B43] Nielsen E. W. Hellerud B. C. Thorgersen E. B. Castellheim A. Pharo A. Lindstad J.. (2009). A new dynamic porcine model of meningococcal shock. Shock 32 (3), 302–309. doi: 10.1097/SHK.0b013e31819c37be 19174740

[B44] Oldrini D. Fiebig T. Romano M. R. Proietti D. Berger M. Tontini M.. (2018). Combined chemical synthesis and tailored enzymatic elongation provide fully synthetic and conjugation-ready neisseria meningitidis serogroup X vaccine antigens. ACS Chem. Biol. 13 (4), 984–994. doi: 10.1021/acschembio.7b01057 29481045

[B45] Opal S. M. (1999). “"The value of animal models in endotoxin research,",” in Endotoxin in health and disease. Eds. Brade H. Opal S. M. (New York, Basel: Marcel Dekker), 809–816. V.S. N. & M.D. C.

[B46] Osterud B. Flaegstad T. (1983). Increased tissue thromboplastin activity in monocytes of patients with meningococcal infection: related to an unfavourable prognosis. Thromb. Haemost. 49 (1), 5–7. doi: 10.1055/s-0038-1657303 6845273

[B47] Ovstebø R. Olstad O. K. Brusletto B. Møller A. S. Aase A. Haug K. B.. (2008). Identification of genes particularly sensitive to lipopolysaccharide (LPS) in human monocytes induced by wild-type versus LPS-deficient neisseria meningitidis strains. Infect. Immun. 76 (6), 2685–2695. doi: 10.1128/iai.01625-07 18362127 PMC2423066

[B48] Ovstebo R. Brandtzaeg P. Brusletto B. Haug K. B. Lande K. Hoiby E. A.. (2004). Use of robotized DNA isolation and real-time PCR to quantify and identify close correlation between levels of neisseria meningitidis DNA and lipopolysaccharides in plasma and cerebrospinal fluid from patients with systemic meningococcal disease. J. Clin. Microbiol. 42 (7), 2980–2987. doi: 10.1128/jcm.42.7.2980-2987.2004 15243048 PMC446236

[B49] Piet J. R. Veld R. A. G. H. in 't van Schaik B. D. van Kampen A. H. Baas F. van de Beek D.. (2011). Genome sequence of neisseria meningitidis serogroup b strain H44/76. J. Bacteriol. 193 (9), 2371–2372. doi: 10.1128/jb.01331-10 21378179 PMC3133077

[B50] Riordan A. Marzouk O. Thomson A. P. J. Sills J. A. Hart A. C. (2002). Prospective validation of the Glasgow meningococcal septicaemia prognostic score. comparison with other scoring methods. Eur. J. Pediatr. 161, 531–537. doi: 10.1007/s00431-002-1024-7 12297899

[B51] Rosenstein N. E. Perkins B. A. Stephens D. S. Popovic T. Hughes J. M. (2001). Meningococcal disease. N. Engl. J. Med. 344 (18), 1378–1388. doi: 10.1056/nejm200105033441807 11333996

[B52] Scrivano S. Sanavio M. Tozzo P. Caenazzo L. (2019). Analysis of RNA in the estimation of post-mortem interval: a review of current evidence. Int. J. Legal. Med. 133 (6), 1629–1640. doi: 10.1007/s00414-019-02125-x 31317317

[B53] Steeghs L. den Hartog R. den Boer A. Zomer B. Roholl P. van der Ley P. (1998). Meningitis bacterium is viable without endotoxin. Nature 392 (6675), 449–450. doi: 10.1038/33046 9548250

[B54] Stephens D. S. Greenwood B. Brandtzaeg P. (2007). Epidemic meningitis, meningococcaemia, and neisseria meningitidis. Lancet 369 (9580), 2196–2210. doi: 10.1016/s0140-6736(07)61016-2 17604802

[B55] van Deuren M. (1994). Kinetics of tumour necrosis factor-alpha, soluble tumour necrosis factor receptors, interleukin 1-beta and its receptor antagonist during serious infections. Eur. J. Clin. Microbiol. Infect. Dis. 13 Suppl 1, S12–S16. doi: 10.1007/bf02390680 7821299

[B56] van Deuren M. Brandtzaeg P. van der Meer J. W. (2000). Update on meningococcal disease with emphasis on pathogenesis and clinical management. Clin. Microbiol. Rev. 13 (1), 144–166. doi: 10.1128/CMR.13.1.144 10627495 PMC88937

[B57] Vogel C. Marcotte E. M. (2012). Insights into the regulation of protein abundance from proteomic and transcriptomic analyses. Nat. Rev. Genet. 13 (4), 227–232. doi: 10.1038/nrg3185 22411467 PMC3654667

[B58] Waage A. Brandtzaeg P. Halstensen A. Kierulf P. Espevik T. (1989). The complex pattern of cytokines in serum from patients with meningococcal septic shock. association between interleukin 6, interleukin 1, and fatal outcome. J. Exp. Med. 169 (1), 333–338. doi: 10.1084/jern.169.1.333 2783334 PMC2189201

[B59] Wright D. O. Reppert L. B. (1946). Fulminating meningococcemia with vascular collapse (Waterhouse-friderichsen syndrome); report on four adult patients who recovered. Arch. Intern. Med. (Chic). 77 (2), 143–150. doi: 10.1001/archinte.1946.00210370024003 21018881

[B60] Zhu Y. Wang L. Yin Y. Yang E. (2017). Systematic analysis of gene expression patterns associated with postmortem interval in human tissues. Sci. Rep. 7 (1), 5435. doi: 10.1038/s41598-017-05882-0 28710439 PMC5511187

