# Supplementary material for: Transcriptomic changes in the large organs in lethal meningococcal shock are reflected in a porcine shock model
Source: Front Cell Infect Microbiol. 2022 Aug 11;12:908204. doi: 10.3389/fcimb.2022.908204 (PMC9413276; doi:10.3389/fcimb.2022.908204)
Supplement: Supplementary Figure 1_1 — Transcriptional profiles of canonical pathways in FFPE tissue samples from meningococcal septic shock patients. The figure displays the top canonical pathways enriched in each organ. A ≪core analysis≫ with FC ≥ |2.0| and p-value < 0.05 was performed separately for each organ. Significantly enriched canonical pathways were identified with a right-tailed Fisher’s exact test p < 0.05, after correction for multiple testing using the Benjamini-Hochberg method. The Z-score = | ± 2| indicates predicted activation state of canonical pathway. Blue color or lighter shades of blue indicate a negative Z-score and down-regulation of the pathway, and orange or lighter shades of orange indicate a positive Z-score and up-regulation of the pathway. Gray color indicates no activity pattern available. Z-score value >| ± 2| are displayed. Z-score limit 2 for lungs, heart, and kidneys. For liver, Z-score 1, and for spleen, no Z-score limit. Pathways that are not involved for that particular organ or disease are filtered away. [file DataSheet_1.zip › Additional file 4_ Table 2.pdf]

© 2000-2022 QIAGEN. All rights reserved.	FC-value	FC-value	FC-value	FC-value	FC-value	FC-value	FC-value	FC-value	FC-value
**Genes in the LXR/RXR Activation network**	Lungs Human	Heart Human	Kidneys Human	Liver Human	Spleen Human	Lungs Porcine	Kidneys Porcine	Liver Porcine	Spleen Porcine
IL6	3,5	2,4	2,3	1,5	1,4	145,2	59,4	65,3	90,4
IL1B	3,1	1,7	1,1	1,6	2,1	98,9	39,4	68,3	55,8
CCL2	10,1	8,4	5,5	2,5	4,1	39,4	69,0	33,1	26,8
IL1A	N/A	N/A	N/A	N/A	N/A	14,1	11,5	12,2	14,6
IL1RN	N/A	N/A	N/A	N/A	N/A	10,1	3,8	7,3	7,1
IL1RL1	4,7	8,3	10,5	1,4	2,1	N/A	N/A	N/A	N/A
S100A8	1,8	2,8	2,2	2,7	1,6	3,6	2,7	2,7	2,7
LDLR	1,9	2,3	1,5	1,6	1,2	1,8	3,5	1,5	4,2
TNF	N/A	N/A	N/A	N/A	N/A	4,7	2,8	3,2	5,2
SAA1	3,8	1,3	2,8	5,1	1,9	N/A	N/A	N/A	N/A
ABCA1	1,6	1,5	1,5	1,2	1,6	2,1	1,5	1,5	2,0
LBP	1,0	-1,2	1,6	1,6	1,3	1,6	13,6	-5,1	-1,1
IL18	N/A	N/A	N/A	N/A	N/A	4,9	3,1	2,2	2,6
TNFRSF1A	1,3	1,4	1,8	-1,2	1,5	1,7	2,7	1,5	1,2
C3	2,2	1,5	3,7	-1,7	1,4	1,2	2,6	-1,9	1,9
HMGCR	2,0	1,7	2,0	3,0	1,4	N/A	N/A	N/A	N/A
RELA	1,6	1,9	2,1	1,3	3,3	N/A	N/A	N/A	N/A
LYZ	-1,1	1,4	1,3	-1,1	1,2	1,6	1,8	2,3	1,2
SAA2	1,6	1,1	1,4	3,2	1,1	N/A	N/A	N/A	N/A
NFKB2	1,9	1,6	1,7	1,6	1,4	N/A	N/A	N/A	N/A
TLR4	N/A	N/A	N/A	N/A	N/A	2,3	3,2	3,3	-1,5

© 2000-2022 QIAGEN. All rights reserved.	FC-value	FC-value	FC-value	FC-value	FC-value	FC-value	FC-value	FC-value	FC-value
**Genes in the PPAR Signaling network**	Lungs Human	Heart Human	Kidneys Human	Liver Human	Spleen Human	Lungs Porcine	Kidneys Porcine	Liver Porcine	Spleen Porcine
IL1B	3,1	1,7	1,1	1,6	2,1	98,9	39,4	68,3	55,8
NFKBIA	3,8	2,7	3,0	2,3	4,7	4,6	14,9	13,1	6,9
IL1A	N/A	N/A	N/A	N/A	N/A	14,1	11,5	12,2	14,6
FOS	1,7	2,1	2,3	1,9	1,5	5,4	8,3	2,5	4,5
IL1RN	N/A	N/A	N/A	N/A	N/A	10,1	3,8	7,3	7,1
IL1RL1	4,7	8,3	10,5	1,4	2,1	N/A	N/A	N/A	N/A
STAT5A	N/A	N/A	N/A	N/A	N/A	4,1	6,1	9,2	4,8
HSP90AA1	1,7	3,5	4,1	2,0	5,1	1,5	1,8	1,7	1,5
TNF	N/A	N/A	N/A	N/A	N/A	4,7	2,8	3,2	5,2
HSP90B1	1,7	2,4	3,2	2,1	4,1	1,3	1,3	-1,4	1,1
MAP2K1	1,7	1,6	1,4	1,3	1,3	1,7	1,7	2,1	1,1
HSP90AB1	1,5	2,7	3,0	1,5	4,2	N/A	N/A	N/A	N/A
IL18	N/A	N/A	N/A	N/A	N/A	4,9	3,1	2,2	2,6
TNFRSF1A	1,3	1,4	1,8	-1,2	1,5	1,7	2,7	1,5	1,2
RELA	1,6	1,9	2,1	1,3	3,3	N/A	N/A	N/A	N/A
RASD1	1,4	1,0	3,2	2,8	1,3	N/A	N/A	N/A	N/A
RAP2A	N/A	N/A	N/A	N/A	N/A	1,2	2,2	3,4	1,5

© 2000-2022 QIAGEN. All rights reserved.	FC-value	FC-value	FC-value	FC-value	FC-value	FC-value	FC-value	FC-value	FC-value
**Genes in the PPARα/RXRα Activation network**	Lungs Human	Heart Human	Kidneys Human	Liver Human	Spleen Human	Lungs Porcine	Kidneys Porcine	Liver Porcine	Spleen Porcine
IL6	3,5	2,4	2,3	1,5	1,4	145,2	59,4	65,3	90,4
IL1B	3,1	1,7	1,1	1,6	2,1	98,9	39,4	68,3	55,8
NFKBIA	3,8	2,7	3,0	2,3	4,7	4,6	14,9	13,1	6,9
IL1RL1	4,7	8,3	10,5	1,4	2,1	N/A	N/A	N/A	N/A
TGFB3	1,4	-1,1	-1,1	1,3	1,1	4,2	2,5	12,3	4,6
HSP90AA1	1,7	3,5	4,1	2,0	5,1	1,5	1,8	1,7	1,5
PRKAR1A	2,1	2,0	3,8	1,3	7,3	1,2	1,1	1,4	1,3
TGFB1	1,8	1,2	1,6	1,3	1,1	1,9	2,8	3,8	2,7
SMAD3	1,6	1,6	2,5	1,3	1,4	2,2	1,6	1,4	3,1
HSP90B1	1,7	2,4	3,2	2,1	4,1	1,3	1,3	-1,4	1,1
ABCA1	1,6	1,5	1,5	1,2	1,6	2,1	1,5	1,5	2,0
MAP2K1	1,7	1,6	1,4	1,3	1,3	1,7	1,7	2,1	1,1
GK	1,6	1,2	1,3	1,4	1,5	2,3	1,1	1,6	1,3
HSP90AB1	1,5	2,7	3,0	1,5	4,2	N/A	N/A	N/A	N/A
JAK2	N/A	N/A	N/A	N/A	N/A	3,6	1,9	3,9	3,4
SMAD4	1,3	1,3	1,6	1,1	1,7	1,1	1,0	1,2	1,1
PDIA3	1,7	2,1	2,5	1,7	2,5	N/A	N/A	N/A	N/A
RELA	1,6	1,9	2,1	1,3	3,3	N/A	N/A	N/A	N/A
RASD1	1,4	1,0	3,2	2,8	1,3	N/A	N/A	N/A	N/A
RAP2A	N/A	N/A	N/A	N/A	N/A	1,2	2,2	3,4	1,5

© 2000-2022 QIAGEN. All rights reserved.	FC-value	FC-value	FC-value	FC-value	FC-value	FC-value	FC-value	FC-value	FC-value
**Genes in the NF-κB Signaling network**	Lungs Human	Heart Human	Kidneys Human	Liver Human	Spleen Human	Lungs Porcine	Kidneys Porcine	Liver Porcine	Spleen Porcine
IL1B	3,1	1,7	1,1	1,6	2,1	98,9	39,4	68,3	55,8
CD40	1,4	1,4	1,5	1,1	1,2	10,2	17,9	20,5	7,1
NFKBIA	3,8	2,7	3,0	2,3	4,7	4,6	14,9	13,1	6,9
IL1A	N/A	N/A	N/A	N/A	N/A	14,1	11,5	12,2	14,6
TNFAIP3	10,9	5,2	4,2	2,4	8,8	N/A	N/A	N/A	N/A
IL1RN	N/A	N/A	N/A	N/A	N/A	10,1	3,8	7,3	7,1
TNIP1	2,4	1,9	1,9	1,7	1,8	3,7	2,5	3,2	4,7
TLR2	1,6	1,1	1,2	1,4	1,2	2,7	3,9	3,1	2,7
TNF	N/A	N/A	N/A	N/A	N/A	4,7	2,8	3,2	5,2
GSK3B	1,6	1,3	1,8	1,5	2,3	1,9	1,7	1,1	1,9
BMP2	N/A	N/A	N/A	N/A	N/A	4,2	1,4	4,0	5,2
IL18	N/A	N/A	N/A	N/A	N/A	4,9	3,1	2,2	2,6
TNFRSF1A	1,3	1,4	1,8	-1,2	1,5	1,7	2,7	1,5	1,2
FCER1G	2,7	2,8	2,0	2,6	2,2	-1,2	-1,0	1,4	-1,2
RELA	1,6	1,9	2,1	1,3	3,3	N/A	N/A	N/A	N/A
IGF2R	1,6	1,4	1,8	1,0	1,5	1,0	-1,1	1,3	1,3
RASD1	1,4	1,0	3,2	2,8	1,3	N/A	N/A	N/A	N/A
IRAK3	2,9	1,6	1,2	2,0	1,8	N/A	N/A	N/A	N/A
IGF1R	1,2	1,4	1,3	1,3	1,2	1,2	1,0	-1,1	1,2
PIK3R5	N/A	N/A	N/A	N/A	N/A	2,0	1,4	2,8	2,6
RAP2A	N/A	N/A	N/A	N/A	N/A	1,2	2,2	3,4	1,5
NFKB2	1,9	1,6	1,7	1,6	1,4	N/A	N/A	N/A	N/A
EIF2AK2	2,1	1,4	1,3	1,0	2,3	N/A	N/A	N/A	N/A
CASP8	N/A	N/A	N/A	N/A	N/A	1,2	2,9	2,4	1,4
MYD88	N/A	N/A	N/A	N/A	N/A	1,8	1,9	2,2	1,9
MAP4K4	1,7	1,6	1,7	1,3	1,4	N/A	N/A	N/A	N/A
CREBBP	1,5	1,5	1,7	1,3	1,5	N/A	N/A	N/A	N/A
TLR4	N/A	N/A	N/A	N/A	N/A	2,3	3,2	3,3	-1,5

Additional file 4_Table 2

Genes in predicted signaling pathways from the top downregulated canonical pathways in FFPE tissue samples from patients with meningococcal septic shock and in organs from porcinis infused with exponentially increasing numbers of *N. meningitidis* (reference strain H44/76) vs. controls.

The genes in the gene signaling network are expressed as Fold Change (FC) values.

Note that only genes from the top downregulated canonical pathways are shown. N/A =not applicable.
