# Supplementary figures and images for: Transcriptomic changes in the large organs in lethal meningococcal shock are reflected in a porcine shock model
Source: Front Cell Infect Microbiol. 2022 Aug 11;12:908204. doi: 10.3389/fcimb.2022.908204 (PMC9413276; doi:10.3389/fcimb.2022.908204)

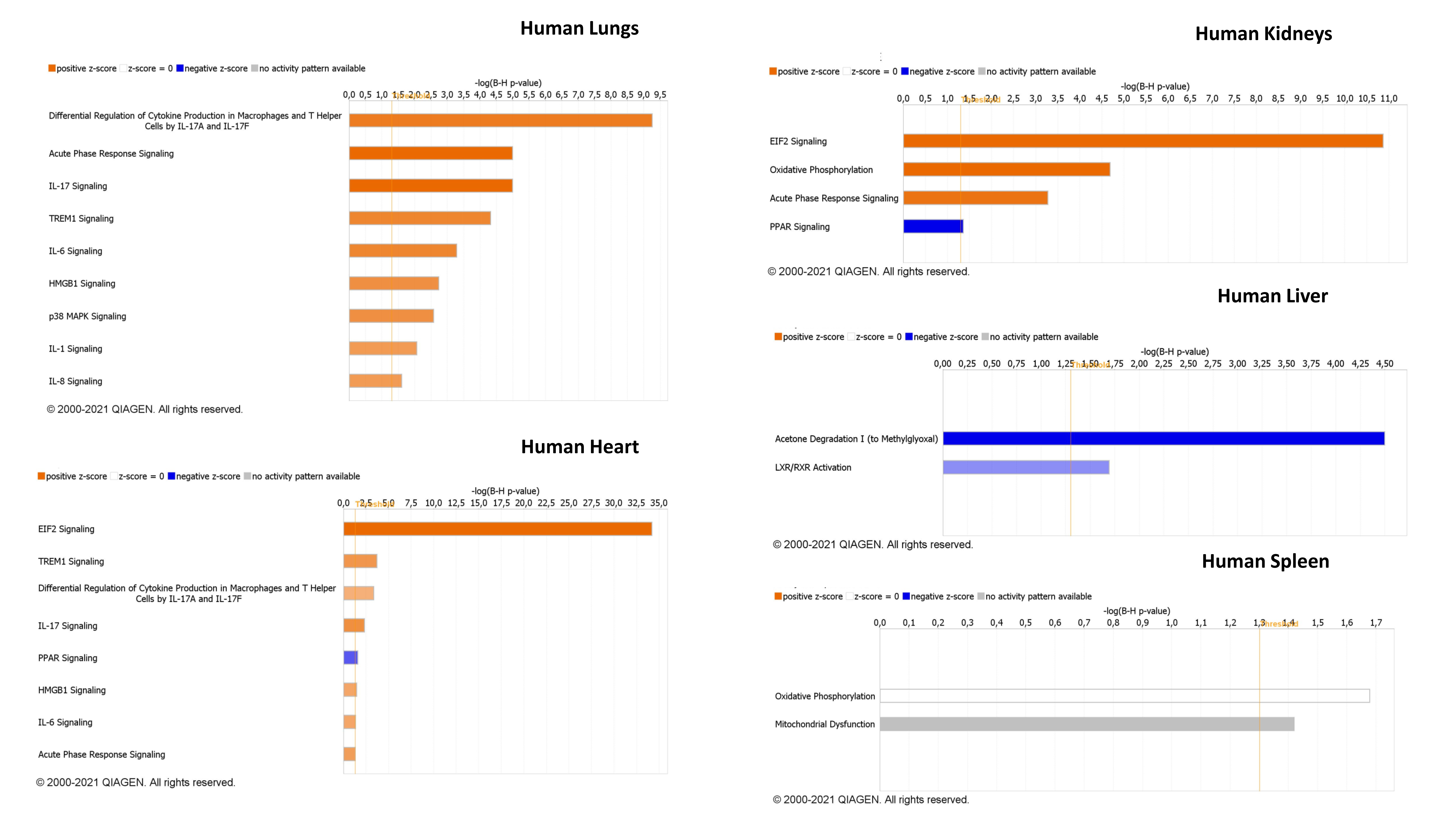

Supplement: Supplementary Figure 1_1 — Transcriptional profiles of canonical pathways in FFPE tissue samples from meningococcal septic shock patients. The figure displays the top canonical pathways enriched in each organ. A ≪core analysis≫ with FC ≥ |2.0| and p-value < 0.05 was performed separately for each organ. Significantly enriched canonical pathways were identified with a right-tailed Fisher’s exact test p < 0.05, after correction for multiple testing using the Benjamini-Hochberg method. The Z-score = | ± 2| indicates predicted activation state of canonical pathway. Blue color or lighter shades of blue indicate a negative Z-score and down-regulation of the pathway, and orange or lighter shades of orange indicate a positive Z-score and up-regulation of the pathway. Gray color indicates no activity pattern available. Z-score value >| ± 2| are displayed. Z-score limit 2 for lungs, heart, and kidneys. For liver, Z-score 1, and for spleen, no Z-score limit. Pathways that are not involved for that particular organ or disease are filtered away. [file DataSheet_1.zip › Additional file 1_Figure S1.tif]

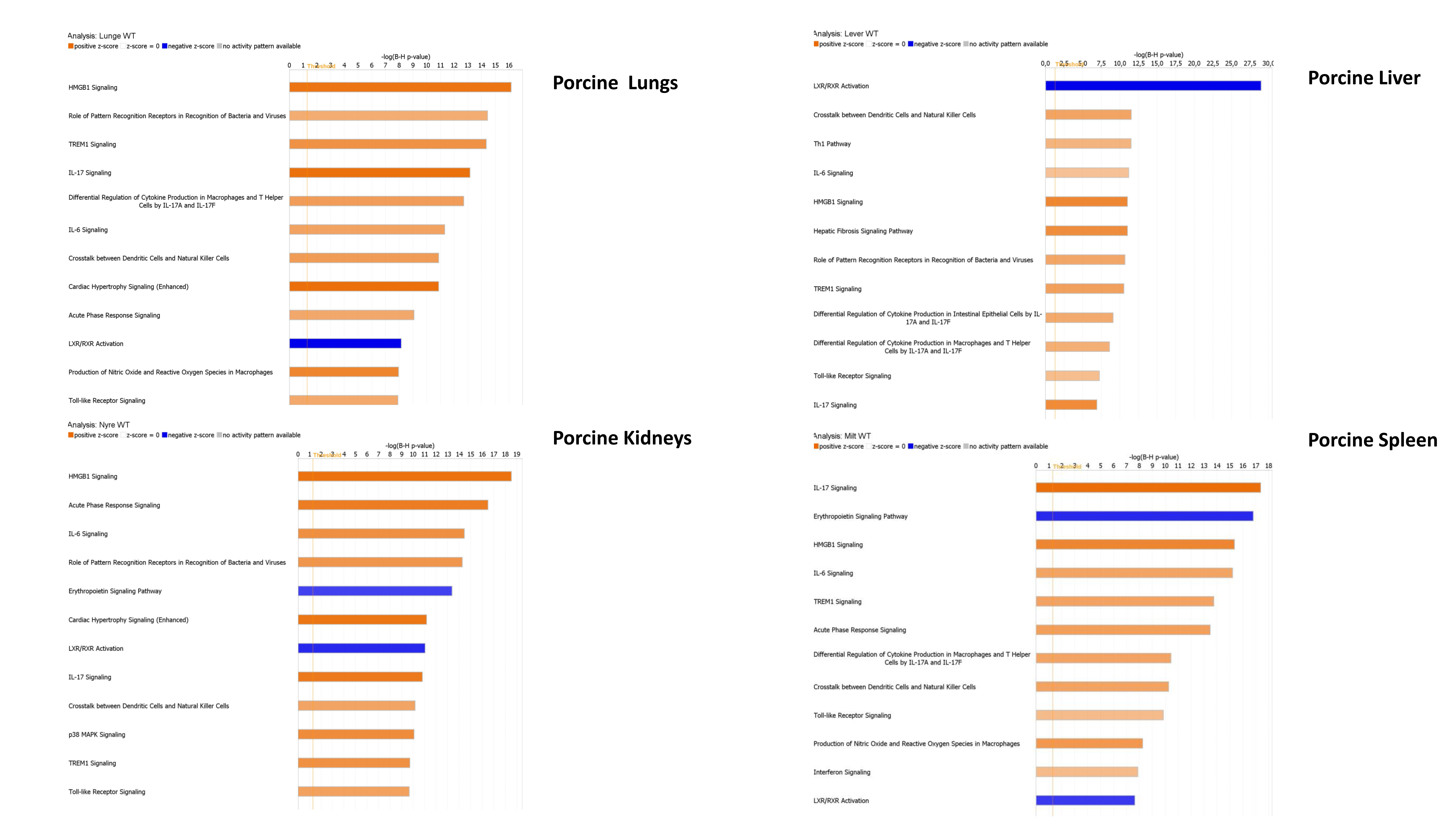

Supplement: Supplementary Figure 1_1 — Transcriptional profiles of canonical pathways in FFPE tissue samples from meningococcal septic shock patients. The figure displays the top canonical pathways enriched in each organ. A ≪core analysis≫ with FC ≥ |2.0| and p-value < 0.05 was performed separately for each organ. Significantly enriched canonical pathways were identified with a right-tailed Fisher’s exact test p < 0.05, after correction for multiple testing using the Benjamini-Hochberg method. The Z-score = | ± 2| indicates predicted activation state of canonical pathway. Blue color or lighter shades of blue indicate a negative Z-score and down-regulation of the pathway, and orange or lighter shades of orange indicate a positive Z-score and up-regulation of the pathway. Gray color indicates no activity pattern available. Z-score value >| ± 2| are displayed. Z-score limit 2 for lungs, heart, and kidneys. For liver, Z-score 1, and for spleen, no Z-score limit. Pathways that are not involved for that particular organ or disease are filtered away. [file DataSheet_1.zip › Additional file 2_Figure S2.tif]
